# Retinal pigment epithelium-specific CLIC4 mutant is a mouse model of dry age-related macular degeneration

**DOI:** 10.1038/s41467-021-27935-9

**Published:** 2022-01-18

**Authors:** Jen-Zen Chuang, Nan Yang, Nobuyuki Nakajima, Wataru Otsu, Cheng Fu, Howard Hua Yang, Maxwell Ping Lee, Armaan Fazal Akbar, Tudor Constantin Badea, Ziqi Guo, Afnan Nuruzzaman, Kuo-Shun Hsu, Joshua L. Dunaief, Ching-Hwa Sung

**Affiliations:** 1 Department of Ophthalmology, Margaret M. Dyson Vision Research Institute, Weill Cornell Medicine, 1300 York Avenue, New York, NY 10065 USA; 2 The Laboratory of Cancer Biology and Genetics, Center for Cancer Research, National Cancer Institute, National Institutes of Health, Bethesda, MD USA; 3 National Eye Institute, National institute of Health, Bethesda, MD USA; 4 Research and Development Institute, Transilvania University of Brasov, School of Medicine, Brasov, Romania; 5 FM Kirby Center for Molecular Ophthalmology, Scheie Eye Institute, Department of Ophthalmology, Perelman School of Medicine, University of Pennsylvania, Philadelphia, PA USA; 6 Department of Cell and Developmental Biology, Weill Cornell Medicine, 1300 York Avenue, New York, NY 10065 USA; 7 Present Address: Department of Urology, Tokai University, Kanagawa, Japan; 8 Present Address: Department of Biomedical Research Laboratory, Gifu Pharmaceutical University, Gifu, Japan; 9 Present Address: Sloan Kettering Cancer Institute, New York, NY USA

**Keywords:** Macular degeneration, Mechanisms of disease, Visual system

## Abstract

Age-related macular degeneration (AMD) is the leading cause of blindness among the elderly. Dry AMD has unclear etiology and no treatment. Lipid-rich drusen are the hallmark of dry AMD. An AMD mouse model and insights into drusenogenesis are keys to better understanding of this disease. Chloride intracellular channel 4 (CLIC4) is a pleomorphic protein regulating diverse biological functions. Here we show that retinal pigment epithelium (RPE)-specific *Clic4* knockout mice exhibit a full spectrum of functional and pathological hallmarks of dry AMD. Multidisciplinary longitudinal studies of disease progression in these mice support a mechanistic model that links RPE cell-autonomous aberrant lipid metabolism and transport to drusen formation.

## Abstract (web summary)

Age-related macular degeneration (AMD) is a leading cause of blindness and is characterised by the accumulation of lipid deposits, called drusen. Here, the authors show that mice lacking chloride intracellular channel 4 in retinal pigment epithelium have defective lipid processing in the eye and pathological features mirroring human AMD, including drusen formation.

## Introduction

Age-related macular degeneration (AMD) is the most common cause of vision loss in the elderly, with a global prevalence of ~200 million people^1^. Dry AMD is the predominant form of AMD. There is currently no treatment to slow its progression to the advanced form geographic atrophy (GA), which is characterized by the severe loss of retinal pigment epithelium (RPE) cells and photoreceptors. AMD is a complex disease involving both genetic and environmental contributions. The study of human genetics, epidemiology, and the donor eye pathologic investigation has linked AMD to lipid metabolism, lysosomal function, extracellular matrix (ECM) homeostasis, oxidative stress, and inflammation^2–5^. How these disparate pathways are integrated to present the clinical symptoms of AMD clinically is unclear. The lack of a reliable lab animal model is a major hurdle in current AMD research. Several mouse models previously developed based on genetic risks fail to fully recapitulate the AMD phenotypes, suggesting that genetic and/or pathway redundancy allows for function compensation^6,7^.

The RPE has been long suspected to be the primary lesion site of AMD though other theories exist^8^. The RPE cells form a polarized monolayer between the neural retina and the choroid, serving as the outer blood-retina barrier. Retinal adhesion and retina-RPE crosstalk rely on interdigitation between the RPE microvilli (MV) and the photoreceptor outer segments. The RPE cells, which do not undergo cell division, are responsible for visual pigment regeneration and recycling/degradation of phagocytosed outer segments through the animal’s entire life^9^. The convoluted basal plasma membranes of the RPE cells (basal infoldings) are juxtaposed to the organized ECM Bruch’s membrane (BrM). The BrM modulates the bidirectional material exchange between the RPE and blood circulation through the choriocapillaris. Drusen, lipid-rich deposits within the sub-RPE space and BrM, are the silent hallmark of dry AMD^10–12^. Although the composition of drusen is known^13^, the mechanism that initiates drusen formation and the lipid source of the drusen (RPE-born or blood-born) remains unclear.

Chloride intracellular channel 4 (CLIC4) is in the six-member CLIC family of proteins. Despite the name, its role as a channel remains controversial because crystallographic analyses showed that CLIC4 is a cytosolic protein and has a structure like the antioxidant enzyme omega-type glutathione-S-transferase. Purified CLIC4 recombinant proteins can insert into artificial liposomes in a redox-dependent manner^14,15^. The expression of CLIC4 is influenced by numerous endogenous or environmental factors, including transforming growth factor-beta 1 (TGF-β1), tumor necrosis factor (TNF), vascular endothelial growth factor (VEGF), glucocorticoids, and infection^16–19^. CLIC4 is metamorphic; it was localized to various subcellular compartments, such as the nucleus, cytosol, plasma membrane, and endolysosomes^20–24^. CLIC4 is pleiotropic, participating in a wide range of cellular functions, such as signal transduction, cell adhesion, cell migration, cell death, and gene regulation^16,17,25,26^.

Our lab previously showed that in macrophages CLIC4 has a role in innate immune response^16^. In polarized epithelial cells CLIC4 played a role in endosomal trafficking, cell morphogenesis, and ECM remodeling^22,23^. In rat RPE in vivo, silencing *Clic4* deformed the MVs and basal infoldings and weakened the RPE-retinal adhesion^24^. These and other previous findings prompted us to propose that CLIC4 performs multiple functions required for maintaining RPE homeostasis.

In this work, we show RPE-specific *Clic4* knockout (KO) mice (or RPE^∆*Clic4*^ mice) develop a full spectrum of functional, clinical, and histological hallmarks of dry AMD. Through biomarkers, transcriptomics, bioinformatics, and 3D electron microscopic (EM) analyses, we gained mechanistic insights into the primary subcellular and molecular lesions that drive AMD-like phenotypes. We shed light on the mechanism of drusen-like deposit formation by uncovering the previously unrecognized RPE export of lipid droplets (LDs) into BrM. Our results support a model that mechanistically links RPE cell-autonomous lipid dysregulation to drusen accumulation.

## Results

### RPE^∆*Clic4*^ mice show progressive decline in visual function

We generated the RPE^∆*Clic4*^ mice by multiple crosses between *Best1*-Cre^+/−^ mice and *Clic4* floxed allele homozygotes (*Clic4*^f/f^) (both on rd8-free, C57BL/6 J background). The resulting *Best1*-Cre^+/−^; *Clic4*^f/f^ were referred to as the RPE^∆*Clic4*^ (or KO) mice. Aged-matched *Best1*-Cre^−/−^; *Clic4*^f/f^ control (herein Ctrl), *Best1*-Cre^+/−^; *Clic4*^WT/WT^ (herein CreCtrl) and C57BL6/J wild-type (WT) mice were employed for comparison (Supplementary Table 1). The RPE-specific *Best1* promoter becomes active in ~10–15-day-old mice^27^. We experimentally confirmed the RPE-selective loss of *Clic*4 RNA and protein in 2-month-old (or older) KO mice (see later).

**Fig. 1:**
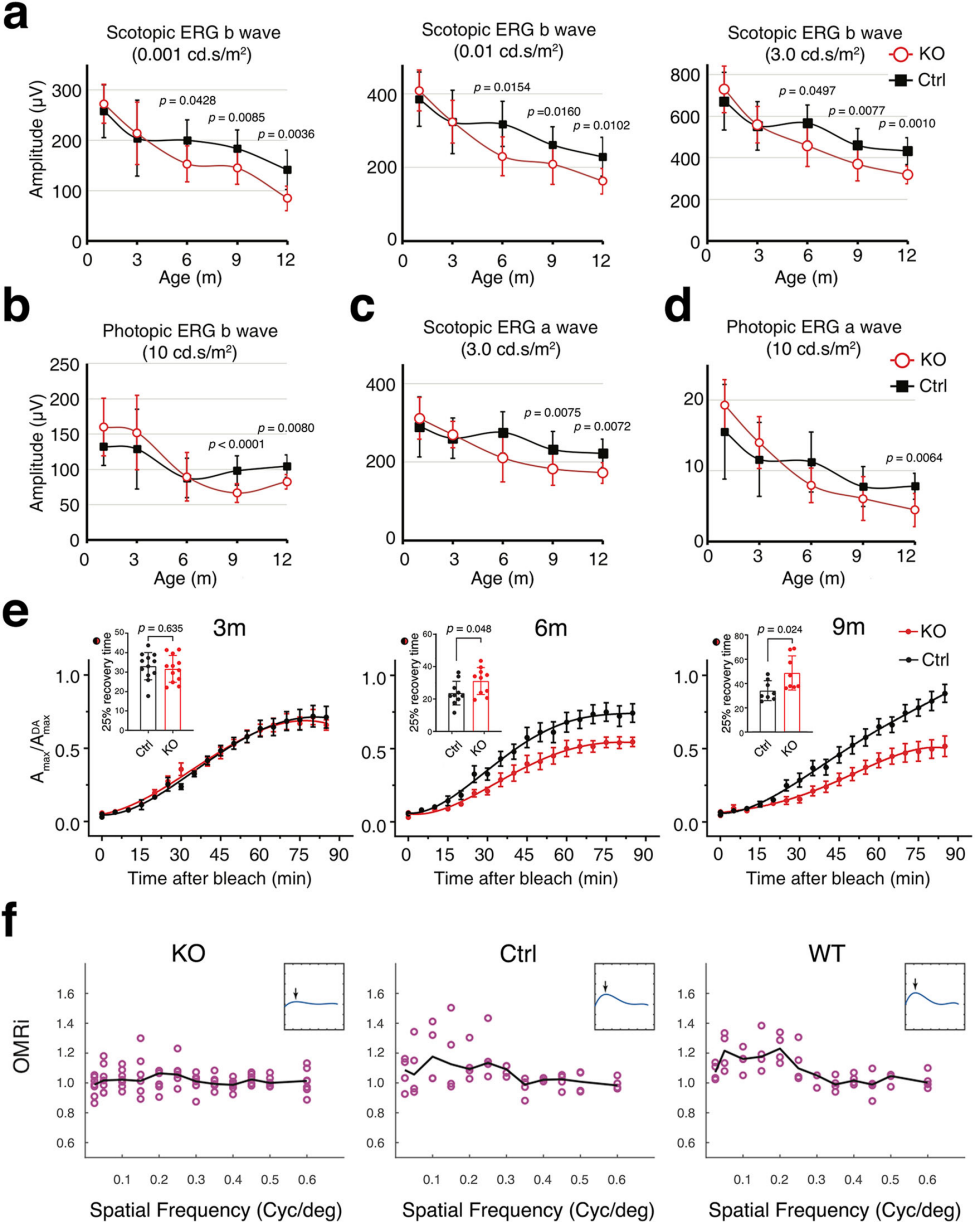
RPE^∆*Clic4*^ mice developed age-related vision loss. **a**–**d** The b-wave (**a**, **b**) or a-wave (**c**, **d**) amplitudes of the scotopic (**a**, **c**) and photopic (**b**, **d**) ERG response evoked by a flash of indicated stimulation intensity are presented. ERG values obtained from Ctrl (*N* = 6, 16, 8, 12, and 8 eyes for 1-, 3-, 6-, 9- and 12-month (m)-old mice respectively) and KO (*N* = 12, 16, 6, 14, and 8 eyes for 1-, 3-, 6-, 9- and 12-month-old mice, respectively) mouse eyes are shown as Means ± SD. Two-tailed student’s *t*-test. **e** KO mice had delayed photobleach recovery. After bleaching >90% of rhodopsin at time 0, the recovery of the scotopic ERG maximal a-wave (*A*_max_) at different time points is shown. Insets show the time required for recovering 25% of *A*_DAmax_ after bleaching. Results obtained from 3-month-old (*N* = 12 eyes), 6-month-old (*N* = 10 eyes), and 9-month-old (*N* = 8 eyes) Ctrl and KO mice are represented as Means ± SEM. Two-tailed student’s *t*-test. **f** OMR index (OMRi) dependency on stimulus spatial frequency, under scotopic conditions, maximal contrast, and optimal stimulus velocity (12 deg/s), for 9 ± 1.5-month-old KO (*N* = 7) and Ctrl (*N* = 4) mice as well as 7 ± 1.5-month-old WT (*N* = 4) mice. OMRi in *y*-axis measures the head movements in correct/incorrect direction. Cycles (cyc)/degree (deg) on the *x*-axis is a measure of spatial frequency. Black lines represent medians across all mice. Insets represent fourth-order polynomial fits, at the same scale as the original data. Arrows point at optimal spatial frequency. Source data of (**a**–**f**) are provided as Source Data.

In human AMD, the rods are more vulnerable than cones^28^. Patients have greater loss of scotopic (vs. photopic) sensitivity and delayed rod-mediated dark adaptation from bright light. In correlation, the rod-mediated scotopic and cone-mediated photopic electroretinogram (ERG) assays showed that the KO mice had the earlier visual function decline in rods than in cones. Compared to the age-matched Ctrl mice (whose ERG also decline during normal aging as described for WT mice^29–31^), the KO mice showed significantly lower b-wave amplitudes of rod (Fig. 1a, Supplementary Fig. 1) and cone (Fig. 1b, Supplementary Fig. 1) ERG beginning at the 6-month and 9-month ages, respectively. The a-wave of the rod (Fig. 1c, Supplementary Fig. 1) and cone (Fig. 1d, Supplementary Fig. 1) ERG signals were detectably lower beginning at the 9-month- and 12-month age, respectively.

To address the dark recovery rate of the KO mice, we measured the rod ERG responses after a bright illumination that bleached >90% of rhodopsin. These studies showed while the 3-month-old KO mice recovered well, the 6- and 9-month-old KO mice had impaired rod-mediated dark adaption (Fig. 1e). It took a significantly longer time for the latter to reach 25% of maximal sensitivity (inset, Fig. 1e).

We next tested the scotopic visual acuity of 9-month-old KO and Ctrl mice using the optomotor response (OMR;^32^ Fig. 1f; Supplementary Fig. 2). Although optimal spatial frequency was similar in KO and Ctrl mice, the optimal response amplitude (OMR index) was modestly affected (median at 0.2 spatial frequency = 1.07 (KO) vs 1.09 (Ctrl), *p* = 0.055, Kolmogorov-Smirnoff test). The mean OMR index of 7-month-old WT mice was 1.23 (Fig. 1f, Supplementary Fig. 2). The spatial frequency threshold for KO mice was 0.51 cycle/degree compared to 0.34 cycle/degree in Ctrl mice. These results overall suggested that the KO mice display visual function defects.

### The clinicopathology of RPE^∆*Clic4*^ mice mimics AMD

**Fig. 2:**
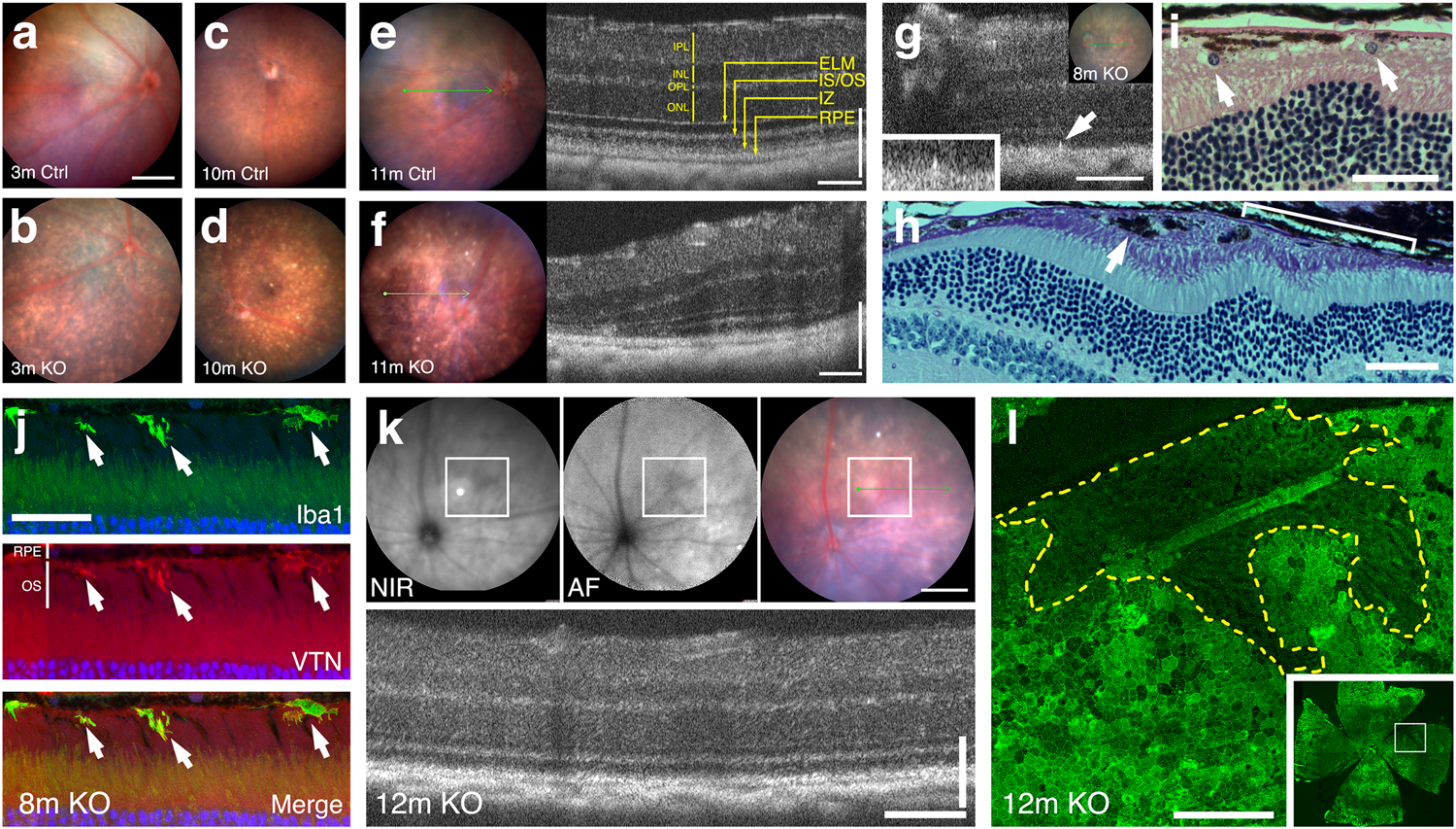
RPE^∆*Clic4*^ mice progressively develop histopathological features resembling intermediate and advanced AMD. **a**–**g** Fundus and OCT images of Ctrl (**a**, **c**, **e**) and KO (**b**, **d**, **f**, **g**) mice of indicated ages in months (m) are shown. IPL inner plexiform layer, INL inner nuclear layer, OPL outer plexiform layer, ONL outer nuclear layer, ELM external limiting membrane, IS/OS inner segment/outer segment, IZ interdigit zone of RPE/outer segment. **g** Highlights the wedge-shaped hyperreflectivity extending from the RPE toward the outer segment region (arrow, inset). **h**, **i** H&E (**i**) and Toluidine blue (**h**) stained retinal sections >12-month-old KO mice reveal the subretinal expression of pigmented (**h**) and non-pigmented (**i**) cells, as well as the RPE-free zone (bracket). **j** Confocal images show the overlapped Iba1 and vitronectin (VTN) staining distributed between RPE and outer segments (arrows) in a 6-8-month-old KO mouse. **k**, **l** shows the near-infrared (NIR), autofluorescence (AF), color fundus and OCT images (in **k**) as well as the F-actin stained wholemount (**l**) of the same KO mouse eye. **l** The en face view of the region corresponding to the reduced autofluorescence (boxed in **k** and **l** inset). The enlarged image of this region reveals an RPE-free zone (dashed line) from which the underlying choroidal vessels are visible. Nearby dark patches (due to the RPE cell loss) are also discernable. Representative images of more than three independent experiments (of **a**–**g**; also see Supplementary Table 1) and three independent experiments (of **h**, **i**) are shown. Bars = 400 (fundus photographs in **a**–**f**, **k**), 100 (OCT images in **e**, **f**, **g**, **k**), 50 (**h**), 40 (**I**, **j**), and 200 (**l**) μm.

We used fundoscopy and spectral-domain optical coherence tomography (SD-OCT) to perform a longitudinal examination of the mice, beginning from 2–3-month-old and at intervals of every ~3 months. We found that the 2–3-month-old KO mice already displayed whitish-yellow amorphous lesions (Fig. 2b), which were absent from the Ctrl mice regardless of age (Fig. 2a, c, e, Supplementary Fig. 3a). As time progressed, the fundus lesion size increased and the lesion shape evolved. The fundi eventually had hypo/hyper-pigmented mosaic appearance (Fig. 2d, f, Supplementary Fig. 3b).

The SD-OCT of the KO mice showed retinal lesions around age 6 months. The RPE layers had increased reflectivity. Hyperreflective subretinal foci of variable size and shape were also observed (Fig. 2f, g). Retinal sections stained with histological dye Toluidine blue (Fig. 2h), as well as hematoxylin and eosin (H&E) (Fig. 2i) revealed both non-pigmented and pigmented cells localized in the subretinal space (Fig. 2h, i). The former likely corresponds to the infiltrated CD45^+^/Iba1^+^ macrophages or migrated microglia (Supplementary Fig. 3c). The subretinally-expressed Iba1 signal sometimes extended into the outer segments and had an extensive overlap with vitronectin (Fig. 2j), a feature shared by subretinal drusenoid deposits (aka reticular pseudodrusen) of human AMD eyes^33^. We estimated the number of infiltrating macrophages and/or microglia by counting the circular bright spots in the fundus images (Supplementary Fig. 3d). The quantification studies showed that the macrophages and/or microglia infiltration peaked at 6–9-month of age and then tapered off (Supplementary Fig. 3d). In contrast, the number of subretinally localized pigmented cell bodies, including the RPE cells that have migrated into the subretinal space, significantly increased during aging (Supplementary Fig. 3e).

The SD-OCT and histologically stained retinal sections showed that ~12-month-old or older KO mice had focal thinning of the outer nuclear layer, indicating photoreceptor cell death (Fig. 2f, h, i). KO mice also had the regional loss of RPE cells and possible choroidal vessel abnormalities (Fig. 2h, i, k). Heidelberg imaging revealed the KO mouse fundus exhibited patches of reduced autofluorescence (Fig. 2k). The correlative studies of F-actin-stained RPE whole mounts suggested that the reduced fundus autofluorescence corresponded to areas having massive RPE cell death (Fig. 2l). Large RPE-free zones revealed the underlying choroidal vessels (dashed lines; Fig. 2l). Small RPE-free patches were also broadly observed. Thus, like human GA, KO mice exhibited severe RPE- and photoreceptor cell death.

All the phenotypic changes described above were consistently associated with the KO mice but negligible in the aged-matched Ctrl (Supplementary Figs. 1, 2, 3a, 3f), CreCtrl (Supplementary Figs. 3a, 3g), and WT mice (Supplementary Fig. 2; also see Supplementary Table 1), though there was some degree of variation in terms of phenotypic severity between KO mice.

### KO mice have early-onset RPE cell morphology alteration and cell death

**Fig. 3:**
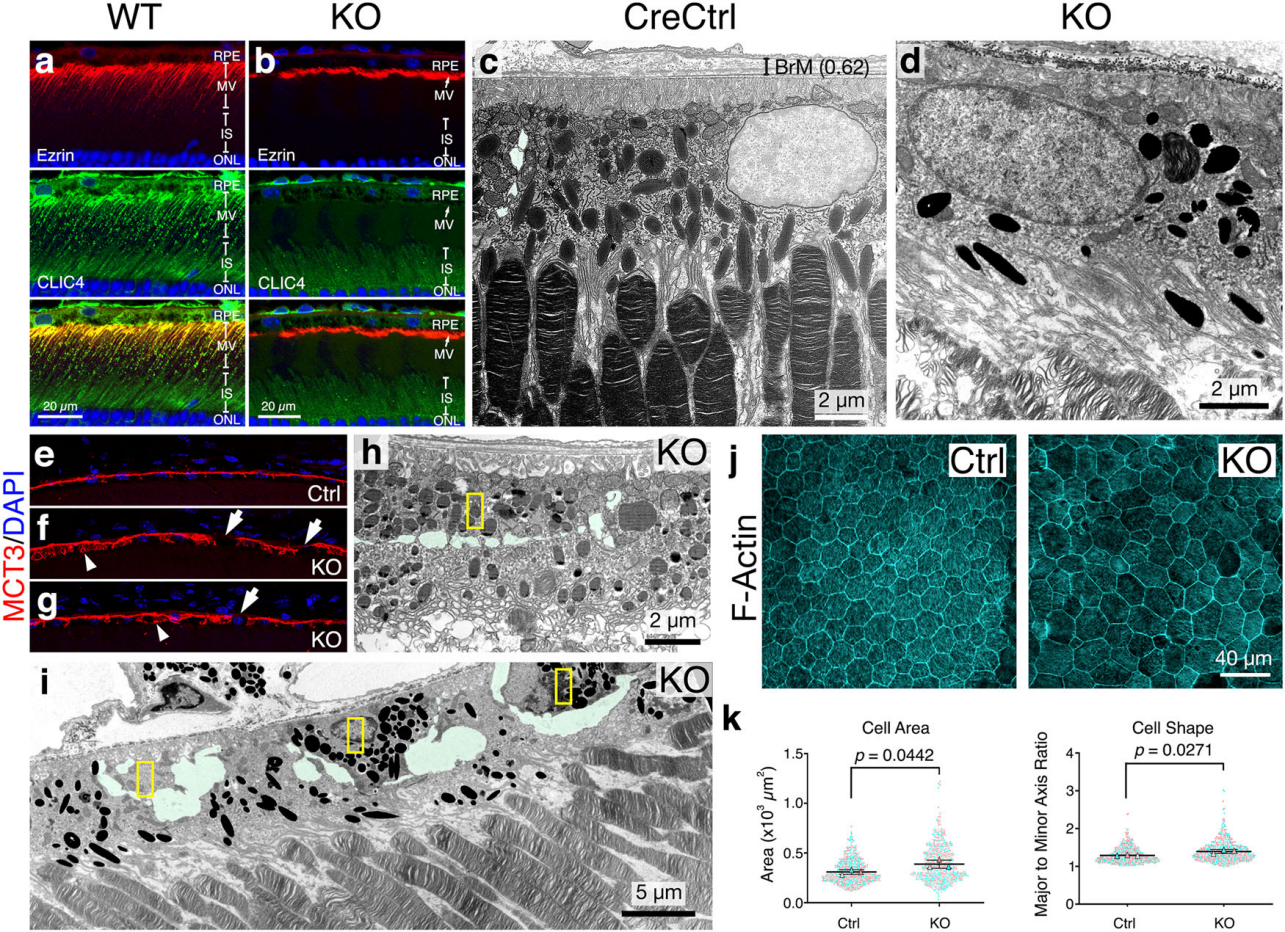
Young RPE^∆*Clic4*^ mice had altered epithelial cell features and increased RPE dropout. **a**, **b** Representative confocal images of 3-month-old WT and KO mouse retinal staining for ezrin and CLIC4. **c**, **d** Representative electron micrographs of 6-month-old CreCtrl (**c**) and 3-month-old KO (**d**) mice. **e**–**g** Representative MCT3 staining of 3-month-old Ctrl mice (**e**), as well as 3- (**f**) and 6-month-old (**g**) KO mice. Arrows point to the loss of basal MCT3. Arrowheads marked the abnormal circular signals of MCT3 that indicated the RPE dropouts. **h**, **i** Representative TEM images of 6-month-old KO mouse retinal sections. Yellow asterisks mark the RPE dropouts, which were surrounded by extracellular spaces (highlighted in green). The remaining RPE cells cell bodies have expanded and migrated to cover the RPE-retinal interfaces. **a**–**i** are representative images of three independent experiments. **j** Representative *en face* views of the 6-month-old Ctrl and KO mouse RPE flat mounts stained for F-actin. **k** Quantification of (**j**). Mean ± SD of the cell area and cell shape of Ctrl and KO mouse RPE cells are shown (600 cells examined over three mouse eyes). Two-tailed Student *t*-test. Source data of (**k**) are provided as a Source Data file.

The retina-RPE-choroid complex of the WT mice broadly expressed CLIC4, albeit its intensity varied among regions (Supplementary Fig. 4a). In the RPE, CLIC4 appeared as bright puncta distributed in the ezrin-labeled RPE microvilli (MVs; Fig. 3a; Supplementary Figs. 4a–b). The MV-expressed CLIC4 puncta had an extensive overlap with Lamp2 (a marker for late endosomes, lysosomes^34^) (Supplementary Fig. 4b). In the KO mice, the CLIC4 staining in the RPE largely disappeared, whereas its choroid staining remained. In the CLIC4 deficient RPE cells, the ezrin-labeled MVs collapsed and appeared as an apical “band” (Fig. 3b); the residual Lamp2 was detected at the basal side instead (Supplementary Fig. 4b); These light microscopic changes were concurrent with the loss of CLIC4, happening at ~2–months of age. Transmission EM (TEM) confirmed the deformed MVs and revealed reduced interdigitation between the tilted MVs and outer segments (Fig. 3d vs. c).

The basal side of the RPE cells was also detectably affected in this phase of the disease. The basolateral plasma membrane expression of the monocarboxylate transporter 3 (MCT3 or SLC16A8) was often missing (Fig. 3e–g). The unusual circular-shaped MCT3 staining likely reflects RPE dropout (Fig. 3f, g). Supporting this, TEM revealed abundant dying cells, whose shrunken cell bodies were enveloped by the extracellular space (Fig. 3h, i). Morphologically characteristic autophagosomes were increasingly observed at the basal side of the monolayer (Supplementary Figs. 5a, b). The vacated “apical” spaces resulting from the RPE dropout were covered by the neighboring cells, perhaps through expansion of their cell bodies, modifying their cell–cell junctions, or through cell migration. Furthermore, the deconvolution of the basal infoldings and the enlargement of the extracellular sub-RPE spaces were conspicuous in the KO mice (Supplementary Fig. 5b, see later Fig. 6c–f).

We used the altered cell shape and cell size (via F-actin staining) as surrogates to quantify the RPE dropout in 6-month-old KO mice. The KO (vs. Ctrl) mouse RPE cells had a more variable cell shape and were larger in average (Fig. 3j, k). However, the cell shape and cell size of different regions (temporal, nasal, superior, and inferior) of the KO mouse RPE cells were not significantly different (Supplementary Fig. 5c). We also stained the RPE sheets for Cre and demonstrated its broad expression in the KO mice of 3-month-olds (Supplementary Fig. 5d).

### Transcriptional reprogramming caused by CLIC4 deficiency mimics AMD risks

**Fig. 4:**
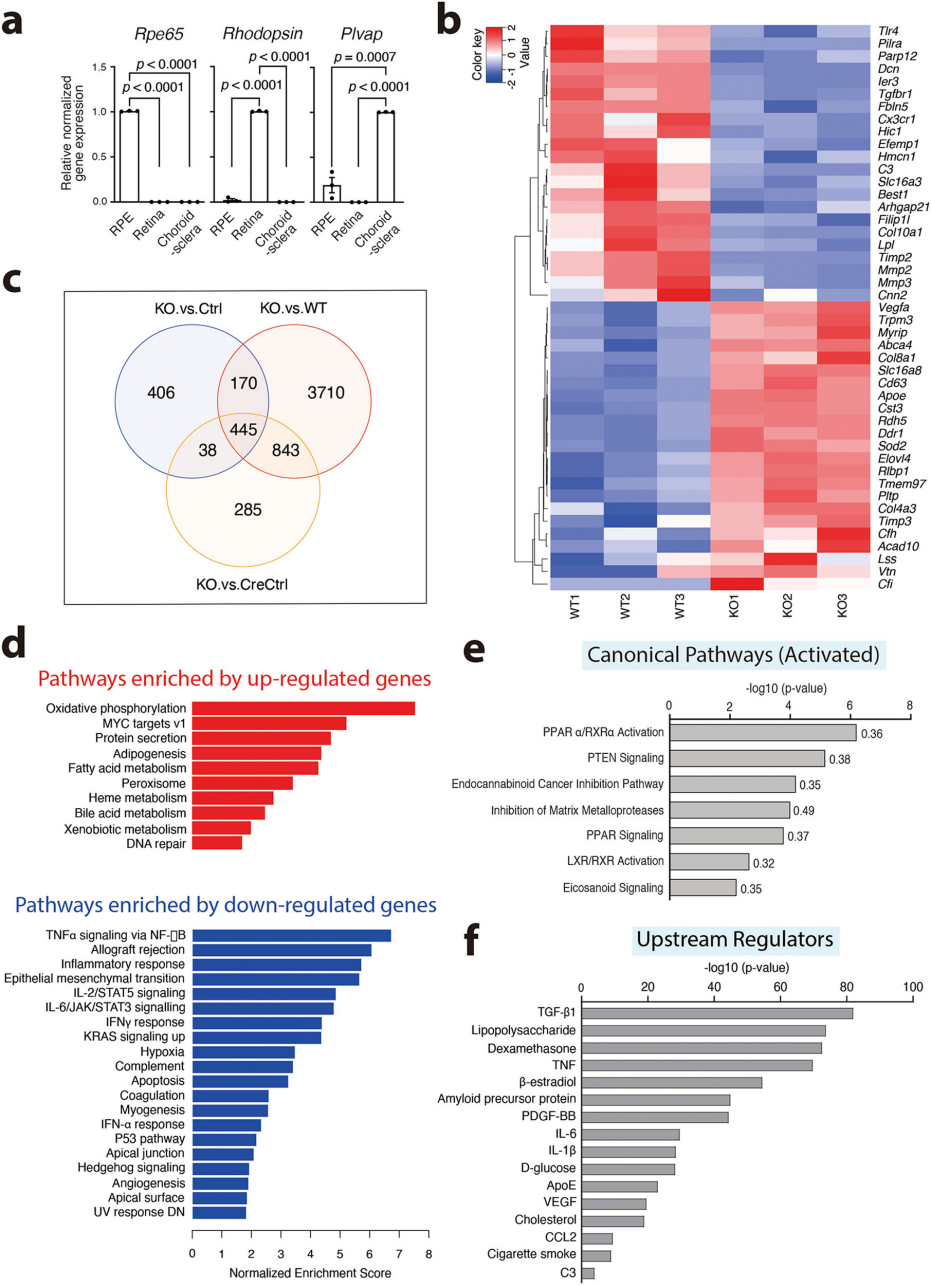
CLIC4 deficiency causes transcriptomic reprograming and pathway changes in RPE cells. **a** Tissue-specific RNA enrichment. qPCR results of the indicated genes (Rho: rhodopsin) using RNAs isolated from RPE, retina, or choroid-sclera cups of WT mice as templates. Data are shown as means ± SEM of *n* = 3 biologically independent experiments. Two-tailed Student’s *t*-test. **b** The heat map depicts the AMD risk genes whose expression level was significantly different between the KO and WT mouse RPE cells (*n* = 3 independent experiments). **c** Venn diagrams showing the numbers of three sets of DEGs (FDR < 0.05) from comparisons of KO vs. WT, Ctrl, and CreCtrl. **d** GSEA identified 30 significant Hallmark pathways (FDR < 0.05) in which 10 were upregulated and 20 were downregulated. These pathways were shared by all three groups (KO vs. WT, Cre, CreCtrl). The absolute normalized enrichment scores from the comparison KO vs. CreCtrl are shown. **e**, **f** Examples of IPA-identified enriched activated canonical pathways (**e**) and upstream regulators (**f**). The *P*-values (**e**, **f**) and the ratios (**e**) from the KO vs. WT group are shown. Source data of (**a**, **b**) are provided as a Source Data file.

Next, we performed RNAseq analyses to gain mechanistic insights into the observed RPE lesions. To focus on the early cell-autonomous effects, we isolated RNA from the purified RPE cells of the 3-month-old mice. The RPE purity was supported by the enrichment of RPE65 mRNA using qPCR (Fig. 4a). Conversely, the mRNAs of the rod-expressed protein, rhodopsin, and the choriocapillaris fenestrate expressed protein, plasmalemma vesicle-associated protein (PLVAP), were predominantly expressed in the purified retinas and choroids/sclera, respectively (Fig. 4a). The significant loss of *Clic4* mRNA in the KO RPE cells, compared to either the Ctrl, CreCtrl, or WT was validated by qPCR (Supplementary Fig. 6a).

Our mouse RPE transcriptomes detected the expression of 65 out of 74 reported AMD risk genes. The mouse orthologues of human *CFHR1*, *CFHR3*, and *C20orf85* genes had little or no expression in RPE cells. Six human AMD risk genes (*TNFRSF10A, ARMS2, MMP1, RAX2, PILRB, CETP*) have no mouse orthologues. Interestingly, the expression of 45 risk genes was significantly altered in the KO (vs. WT) RPE cells (adjusted *P*-value (*P*adj) < 0.05). The heat map and the hierarchical clustering reflect these changes (Fig. 4b). These genes encode proteins responsible for diverse functions, such as cell adhesion/cytoskeleton reorganization (ARHGAP21, HMCN1, VTN, CNN2), cell death (IER3, HIC1, DCN), lysosomal function (CST3 CD63), lipid metabolism (ApoE, ELOVL4, LPL, LSS, ACAD10,P LTP, TMEM97), ECM homeostasis (MMP2, MMP3, DCN, COL10A1, COL8A1, COL4A3, TIMP2, TIMP3, DDR1, EFEMP1, FBLN5), innate immunity/inflammation (PILRA, C3, TLR4, CFH, CFI, CX3CR1, VTN, CNN2), visual cycle (RLBP1, RDH5), oxidative stress (SOD2), angiogenesis (VEGFA, FILIP1L), surface signaling/metabolism (BEST1, TRPM2, SLC16A3, SLC16A8, ABCA4, TGFBR1, PARP12), and vesicular trafficking (MYRIP). We used qPCR to confirm the reduced expression of *Mmp2, Dcn*, and *Ler3* in the KO mouse RPE cells (Supplementary Fig. 6b). The transcripts of several cytokines/cytokine receptors previously linked to AMD pathogenesis (e.g., *Ccl2*, *Ccr2*, *Cx3cr1*^35^) were also significantly altered in the KO RPE cells (Supplementary Fig. 6c). These results showed that CLIC4 deficiency had a broad impact on the expression of a cohort of AMD-linked genes.

The three comparisons between the RPE transcriptomes of KO vs. Ctrl, CreCtrl, and WT mice revealed 1059, 1611, and 5168 differentially expressed genes (DEGs; *P* < 0.05, fold change (FC) > 1.5), respectively, and they shared 445 common DEGs (FDR < 0.05) (Fig. 4c). The disparity in the number of DEGs between groups might reflect the dissimilarity of their genetic backgrounds. DAVID analysis of the 445 common DEGs revealed 34 significantly enriched KEGG pathways (*P* < 0.05). Supplementary Fig. 6d showed a partial list of these pathways. Based on the upregulated or downregulated genes in the ranked gene lists, Gene Set Enrichment Analysis (GSEA) identified 30 Hallmark pathways that were significant (FDR < 0.05) and shared among the three groups (Fig. 4d). We also subjected the DEGs of the individual group to Ingenuity Pathway Analysis® (IPA). The three groups shared 154 enriched canonical pathways; Fig. 4e and Supplementary Fig. 6e showed, respectively, examples of the activated and inhibited pathways.

The transcriptome bioinformatics analyses indicate that CLIC4 deficiency affects a myriad of biological processes. These include the pathways modulating oxidative states (e.g., oxidative phosphorylation, hypoxia, peroxisome activity), lipid metabolism (e.g., adipogenesis, fatty acid metabolism), cell-matrix interaction, epithelial-mesenchymal transition (EMT), protein secretion, inflammation (e.g., complement, IL-1, TNF-α/NF-kB, IL-6), apoptosis, signal transduction (e.g., MAPK, TP53), and actin cytoskeleton rearrangement. These results suggest that CLIC4 is a central node that harnesses the complex cellular and signaling pathway networks that maintain RPE homeostasis.

The causal analysis tool of IPA-identified 1,836 upstream regulators shared by all three groups (*p* < 0.05). Several regulators were also the targets whose expression was regulated by CLIC4 (e.g., TGFB1, C3), implicating a strong feedback loop of CLIC4-mediated gene regulation. The best-established AMD environmental risk, cigarette smoke^36^, was identified as an upstream regulator. The top-rank upstream regulator TGF-β1, and several other regulators (e.g., Apolipoprotein E (ApoE), TNF-α, IL-1β, CCL2, C3, VEGF^37^) also had a contributing role to the AMD pathology (Fig. 4f). The resemblance between the signaling pathways and the regulators that conferred the pathology of RPE^∆*Clic4*^ mice and human AMD eyes supports the relevance of RPE^∆*Clic4*^ mice for modeling AMD.

### Dysregulated lipid homeostasis and lipid deposition in CLIC4 deficient RPE

GSEA (Fig. 4d) and IPA (Fig. 4e) consistently revealed that CLIC4 deficient RPE cells had dysregulated lipid metabolism. The latter identified the activation of peroxisome proliferator-activated receptors (PPAR), the liver X receptor (LXR), and the retinoid X receptor (RXR) pathways. These three pathways are the well-established master regulators of lipid metabolism^38^. These proteins form heterodimers upon lipid ligand binding and serve as nuclear transcription factors. The expression of several PPAR/RXR/LXR targets, participating in various aspects of lipid metabolism (e.g., storage, transport, oxidation, synthesis), was altered in the RPE cells (Supplementary Fig. 6f). We were particularly intrigued by the increased expression of two of the targets, perilipin 2 (PLIN2) and ApoE.

**Fig. 5:**
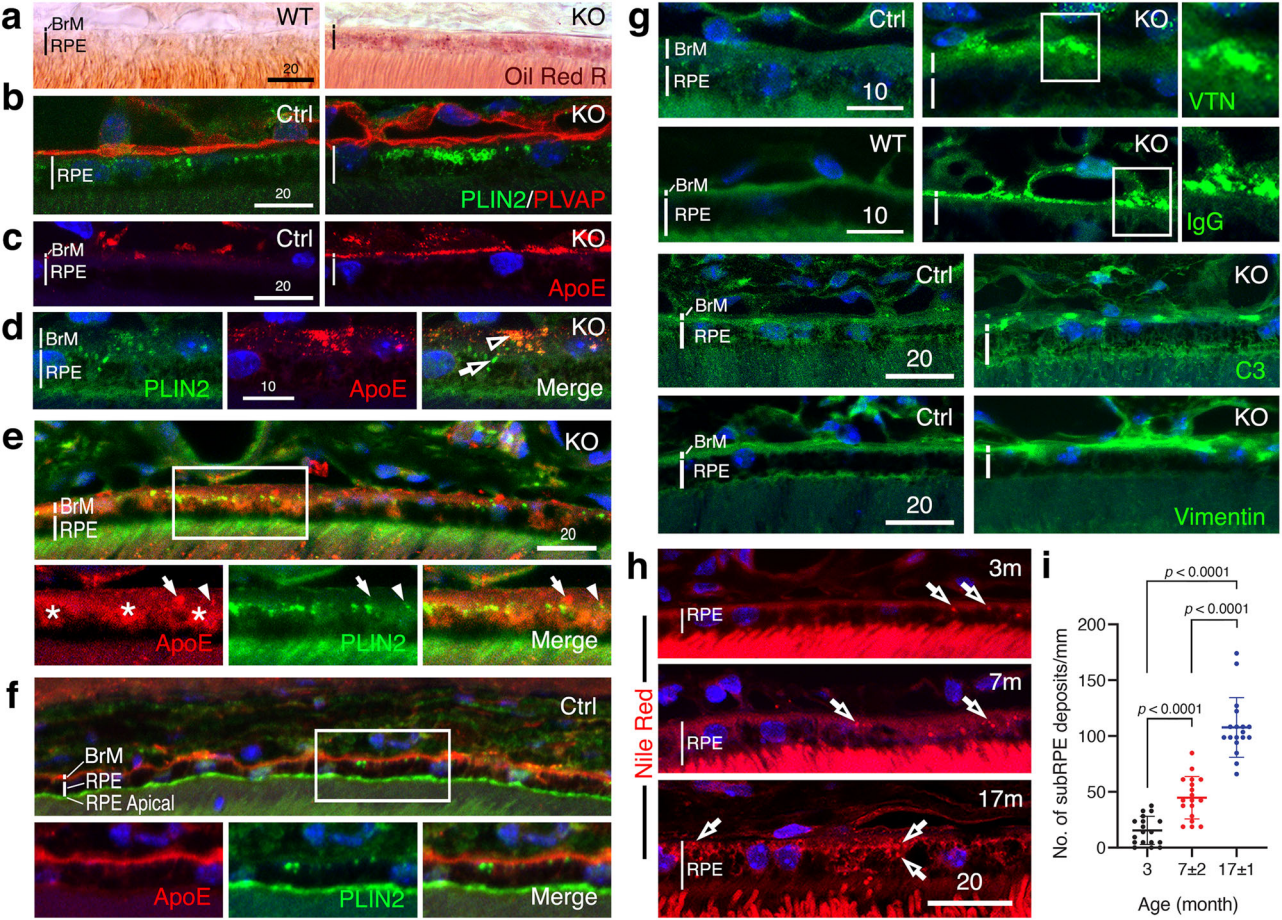
RPE^∆*Clic4*^ mice have aberrant and age-related lipids, lipoproteins, and protein depositions at sub-RPE/BrM. **a**–**d** In 6-8-month-old Ctrl and KO mice, the retina-RPE-choroid complex was stained with lipid dyes (**a**) or antibodies plus DAPI (**b**–**d**). **a** shows Oil Red O staining of RPE-expressed LDs and some BrM areas in a prebleached retina slice. **d** BrM-expressed PLIN2 (arrows) and overlapping ApoE (arrowhead) in a KO mouse retinal slice sectioned tangentially across the RPE-choroid complex. **e**, **f** The RPE-BrM regions of the ~18-month-old KO (**e**) and Ctrl (**f**) mice stained for PLIN2 and ApoE. Bottom panels are the enlarged, boxed areas of the top panels. In (**e**), arrowheads point to the colocalized ApoE and PLIN2; arrows point to the aggregated ApoE signals (arrows); asterisks marked the ApoE-rich sub-RPE deposits. **g** The RPE-BrM region of 10–18-month-old KO (right panels) and Ctrl or WT (left panels) mice stained for the indicated drusen markers. VTN vitronectin, IgG Immunoglobulin. The enlarged boxed areas highlight the aggregated staining pattern of the indicated proteins. **a**–**g** are representative images of three independent experiments. **h** Representative images show the age-dependent increase of Nile Red- stained granular lipid deposits in the basal/sub-RPE regions (open arrows) in the KO mice. **i** Quantification of the sub-RPE granular lipid deposits shown in (**h**). Mean ± SD (18 surveyed areas over *N* = 3 mice) are shown. Two-tailed Student *t*-test. Scale bar in μm. Source data of (**i**) are provided as a Source Data file.

PLIN2 (formerly ADRP) specifically binds to the surface of LDs. Overexpression of PLIN2 causes LD accumulation^39^. We first used the neutral lipid dyes Oil Red R (vs. WT; Fig. 5a) and Nile Red (vs. Ctrl, CreCtrl; Supplementary Fig. 7a), which detect cholesterol esters, to show that the ~6-8-month-old CLIC4 deficient RPE cells had more LDs. Consistently, in either retinal sections (Fig. 5b, Supplementary Fig. 7e) or RPE sheets (Supplementary Figs. 7b–d), PLIN2-labeled LD surface signals were more prominently observed in the KO (vs. Ctrl, CreCtrl) mouse RPE cells.

ApoE, a lipoprotein component, is a major lipid transport protein in the central nervous system. ApoE was localized in the sub-RPE deposits secreted from the basal side of cultured RPE cells^40^ and a component of drusen^41^. While the Ctrl (Fig. 5c, left panel) and CreCtrl (Supplementary Fig. 7e) mouse BrMs expressed little ApoE, the KO mouse BrMs had abundant ApoE (Fig. 5c, right panel). The ApoE in the latter looked aggregated and overlapped with PLIN2 (Fig. 5d). The overlapping ApoE and PLIN2 were particularly evident in the tangential en-face views of the BrMs (i.e., higher spatial resolution). The BrM-expressed aggregates containing both PLIN2 and ApoE, and their overlapping signals further increased in KO mice during aging (Fig. 5e). In contrast, aged Ctrl mice expressed diffuse signals of PLIN2 and ApoE which were largely non-overlapping (Fig. 5f). While ApoE was expressed on the BrM, PLIN2 was primarily expressed on the apical side of the RPE cells.

Compared to the age-matched WT and Ctrl mice, old KO mice also had more abundant expression of several additional drusen components (i.e., vitronectin, immunoglobulin, C3, and vimentin) at the BrM (Fig. 5g). Vitronectin and immunoglobulin were often in aggregates as well. Furthermore, the BrM of the aged KO (vs. Ctrl, CreCtrl) mice had a significantly higher level of lipid deposits (Supplementary Figs. 7f, g). These studies ruled out the possibility that the BrM anomaly could be accounted for by Cre toxicity or senescence alone.

### RPE lipid efflux and lipid sources of drusen

RPE cells have a reverse cholesterol transport function in which they remove excess cholesterol to the choroidal circulation. To date, the mechanism by which lipids efflux from the RPE across the BrM, and the mechanism that underlies drusen formation at the sub-RPE/BrMs are poorly understood. During the process of aging, we found the KO mice had gradually increasing numbers of lipid granules that appeared in the sub-RPE deposits (Fig. 5h, i). To address the link between the lipids localized to basal RPE, sub-RPE, and BrMs, we set out to define the ultrastructural profile of these lipids using TEM and 3D focused-ion beam scanning EM (FIB-SEM). We applied an osmium-based fixation/staining protocol, which has the advantage of differentiating lipids based on their graded electron density (in addition to their shape and size). The FIB-SEM tomogram provides high-solution *en-bloc views* collected through milling the thick tissue blocks at ~20 nm/pixel z-resolution.

**Fig. 6:**
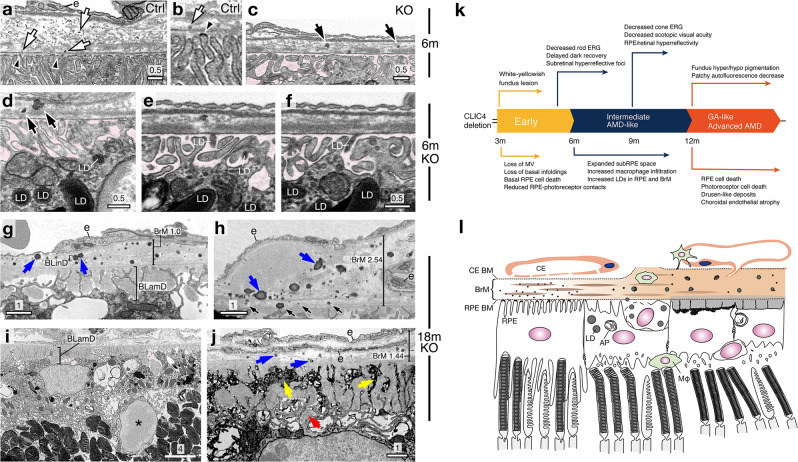
RPE lipid transport, BrM lipid deposition & disease summary for RPE^∆*Clic4*^ mice. **a**, **b** FIB-SEM images of 6-month-old Ctrl mouse RPE-BrM-choroid complex reveal lipoprotein-like particles diffusely expressed in the BrMs (arrows) and emerging from the dark lipid-rafts of the basal plasma membranes (arrowheads). Also see Video 1. **e** Choriocapillaris. **c**–**f** FIB-SEM images of 6-month-old KO mice show the BrM expression of spherical-shape LD-like structures, which are decorated with multiple lipoprotein-like granules (arrows in **c**, **d**). The BrM-expressed LDs shared similar profiles to those detected in the basal RPE cells (**d**) and sub-RPE space (**e**, **f**, highlighted in pink). Also, see Videos 2 and 3. **g**–**j** Representative TEM images of 18-month-old KO mice reveal the BrM-expressed LDs coated with dark particles (blue arrows) and variable size of the lipoprotein-like aggregates. These lipids were frequently seen at the BrM-RPE junction (black arrows in **h**), but not at the BrM-choroid junction. BrMs display dome-shape large deposits (in **h**) and endothelial cells (**e** in **h**, **j**). Labeling indicates the thickness of BrM (in μm), a migratory RPE cell (asterisk in **i**), the membranous debris associated with multi-lobed shape BLamD (yellow arrows in **j**) and long-spaced collagen (red arrows in **j**). Scale bars are in μm. **a**–**j** Representative images of three independent experiments. **k** The temporal order of the AMD-like clinicopathology (top) and histopathology (bottom) manifested in RPE^∆*Clic4*^ mice. **l** A cartoon depicts the retinal-RPE-BrM-choroid complex of a Ctrl mouse (left) and the progressively developed lesions in the RPE^∆*Clic4*^ mice (middle to right). CE choriocapillaris. AP autophagosomes. Mφ macrophages (green). BM basement membranes.

In the young Ctrl mice, abundant fine dark particles were diffusely expressed in the BrMs (Fig. 6a). The granular shape and small size (<30 nm diameter) of these particles are consistent with the morphological characteristics of lipoprotein. These lipoprotein-like particles emerged from lipid raft-like dark patches in RPE basal infoldings (Fig. 6a, b; Video 1). In contrast, age-matched KO mouse RPE cells did not express the lipid rafts on the deconvoluted basal infoldings. The juxtaposed BrM had fewer individual lipoproteins. Instead, the lipoprotein-like particles were attached to LD-like spheres (~100–300 nm diameter; Fig. 6c–f). The Z-stacked tomography revealed that these LD spheres were exported through the sub-RPE spaces from the underlying RPE cells (Fig. 6d–f, Videos 2 and 3), whose basal cytoplasm contained abundant LDs (Fig. 6d–f).

A greater abundance of the similar-looking (shape, size) lipoprotein aggregates, LDs, and lipoprotein-LD hybrids were accumulated in the aged KO mice, forming the drusen-like basal linear deposits (BLinDs) (Fig. 6g, h, j). The aged Ctrl mice did not accumulate BLinDs (Supplementary Fig. 8a). They, as expected^42^, exhibited several age-related changes (e.g., increased lipofuscin, reduced RPE-retina contacts). Taken together with the bioinformatics studies of the young KO mice and the longitudinal studies using lipid dye/lipoprotein biomarkers and EM, these results coherently suggest that RPE cell-autonomous lipid dysregulation predisposes toward the formation of drusen-like deposits. These results further indicate that RPE cells provide the core lipids that accumulate in the drusen-like BLinDs.

Basal laminar deposits (BLamD), which often form a lobular shape, were broadly expressed in the KO mice (Fig. 6g–j). The BLamDs topographically corresponded to the previously expanded sub-RPE spaces, and were filled with grayish deposits, long-spaced collagen, and dark membranous debris (Fig. 6j). Many RPE cells had migrated into the subretinal space; the residual RPE cells had scant internal membranes and cytoplasm (Fig. 6i). The BLinD-encompassing BrMs were thicker (up to ~2.5 μm), calcified, and lacked distinct layered structures. The neighboring choriocapillaris had swollen cell bodies and reduced fenestration. We observed endothelial cells in the BrMs (Fig. 6h, j), indicating potential development of choroidal neovascularization. These results, taken together, showed that the cell-autonomous RPE cell injury produced an AMD-mimicking phenotype spanning the retina-RPE-choroid complexes. They provide support for the long-held theory that RPE can serve as the primary lesion site in AMD.

## Discussion

For a long time, establishing a mouse model of AMD has been an important goal in vision research. Several mouse models with one or multiple AMD-linked genes deleted have been unable to recapitulate the complex disease AMD, perhaps due to gene and/or pathway compensation. A second hit by environmental risks might also be needed to promote the phenotypic manifestation. While several mouse lines have reported AMD-like retinal phenotypes, subsequent studies suggested senescence alone and/or the spontaneous frameshift mutation in the *Crb1* gene *rd8* (that exists in several inbred mouse lines) might contribute to these phenotypes^43–45^. Thus, a genetic mouse model for AMD remains to be demonstrated.

Here we validate the utility of RPE^∆*Clic4*^ mice by showing these mice (on a rd8-free C57BL6/J background) exhibit a full spectrum of functional, clinical, and histological phenotypes resembling dry AMD. RPE^∆*Clic4*^ mice developed each of AMD-like clinicopathologies in a temporal order like the advancement from intermediate to advanced AMD (see the timeline in Fig. 6k). The longitudinal studies showed that several histopathologic phenotypes (e.g., RPE dropout, sub-RPE/BrM lipid deposits, macrophage infiltration) worsen during aging, a hallmark of AMD (summarized in Fig. 6j). Direct-coupled electroretinogram (DC-ERG) signal of 1-month-old KO was comparable to that of Ctrl mice (Supplementary Figs. 8b, c), indicating that the RPE function develops normally in RPE^∆*Clic4*^ mice. Taken together, our results rigorously ruled out the possibility that the observed pathophysiology of the KO mice was due to the potential Cre toxicity, developmental defects, or senescence per se. The RPE^∆*Clic4*^ mice developed extracellular lipid-rich deposits whose geographic location, morphology, and composition resemble human drusen. These mice exhibit age-dependent measurable disease output with high penetrance, which should make them a valuable tool for testing potential therapies for reversing AMD-associated changes.

The stochastic nature of the atrophic areas in the RPE^∆*Clic4*^ mice may simply reflect the lack of a focal area in the mouse with high metabolic load. The human macula may be predisposed to AMD changes because of the high density of photoreceptors increasing the metabolic load on the RPE. But the RPE pigmentary change/atrophy in the retinal periphery of human AMD cases has also been published^46,47^. In AMD within the macula, spatial patterns of drusen and GA can be considered stochastic. Genome-wide association studies have not yet identified *CLIC4* as an AMD risk gene. This is probably because of the importance of CLIC4 in organogenesis^23^. The currently available human “RPE” transcriptomes were obtained from postmortem tissues that included the choroids and sometimes the sclera as well^48–50^. Thus, whether the expression of CLIC4 is altered in the RPE cells of human AMD eyes remains to be determined.

The natural history of fundus changes showed that 2–3-month-old RPE^∆*Clic4*^ mice manifest the first observable abnormality. The amorphous white-yellowish lesions showed up around the time point when the CLIC4 deficient RPE cells exhibited collapsed MVs and reduced interdigitation with the outer segments. Though the attenuated RPE-photoreceptor contacts were readily visible by the ezrin immunostaining and EM, they were not evident in the SD-OCT and H & E-stained paraffin sections. We envision that the chronically reduced contacts between the RPE and photoreceptor outer segments may impair the regeneration of visual pigment (and other outer segment components), which contributes to the delayed dark adaption. The appearance of the fundus lesions gradually evolved. These changes may reflect the accumulation of the lipids in the RPE cells and their underlying sub-RPE/BrMs. Focal demise of photoreceptor and RPE cells might explain the unevenly pigmented appearance of the fundus.

CLIC4 is a pleomorphic protein. The transcriptome/bioinformatic studies showed CLIC4 deficiency had a broad impact on the mRNA levels of several AMD risk genes and a myriad of AMD-associated signaling pathways (e.g., lipid metabolism, ECM, oxidative stress, inflammation, and cell death). The integrated outcomes of these genetic and environmental risks may perturb the biological processes required for maintaining RPE homeostasis. Aging may further exacerbate the maladaptive responses that drive the RPE cells toward cell death. The identification of TGFβ-1 as the top upstream regulator of the CLIC4 deficient RPE is reminiscent of CLIC4 as an integral component of the TGFβ-1 signaling pathway^51^. TGFβ-1 signaling pathways are known to modulate diverse cellular activity^52^. Thus, a portion of the biological processes changed in the CLIC4 deficient RPE cells may be mediated by the TGFβ-1 signaling pathway. The large number of the identified upstream regulators is consistent with the notion that CLIC4 senses danger signals. We propose that further data mining of the upstream regulators may offer additional insights into the risks or the protectors for CLIC4-mediated AMD pathology.

The pathway analyses indicate CLIC4 deficient RPE cells had changed complement pathways and oxidative stress (Fig. 4d, Supplementary Figs. 6d, 6e). Experimentally, we showed that RPE^∆*Clic4*^ (vs. Ctrl) mice had detectably more basal RPE deposition of C3 and C3 activation products (i.e., cleaved fragments C3b/iC3b/C3c) (Supplementary Fig. 9a). The immunostaining (Supplementary Fig. 9b) and ELISA (Supplementary Fig. 9c) assays both showed that the CLIC4 deficient RPE cells expressed more lipid oxidation product 4-hydroxynonenal (4-HNE), a biomarker of oxidative stress. Hyperactivation of complement pathway and oxidative stress have been strongly linked to AMD predisposition^53^. The common signaling pathways and regulators that confer the pathology of RPE^∆*Clic4*^ mice and AMD underscore the relevance of RPE^∆*Clic4*^ mice for investigating the etiology of AMD-like pathophysiology.

Our subcellular and ultrastructural characterizations showed EMT-like features and cell death of RPE cells are among the primary anomalies. EMT was also the most notable cellular event of the RPE cells acutely transfected with *Clic4*-shRNA in situ^24^. Mechanistically, GSEA revealed the enrichment of EMT and apoptosis pathways (Fig. 4d). The IPA-identified inhibition of PTEN pathway (Fig. 4e) is of particular interest. PTEN functionally interacts with CLIC4^23^. PTEN deficient RPE cells underwent concomitant EMT and cell death^54^. The DAVID (Supplementary Fig. 6d) and IPA (Supplementary Fig. 6e) analyses indicated that CLIC4 deficient RPE cells had altered actin cytoskeletons and RhoA GTPase signaling. The activation of RhoA recruits CLIC4 onto the plasma membrane^21^. The plasma membrane-localized CLIC4 can modulate cortical rigidity through modifying the actin-plasma membrane dynamics^55^. We surmise that deregulated cortical actin and rigidity caused by CLIC4 deficiency may disrupt the convoluted membranous structures of MVs and basal infoldings.

Consistent with our previous studies using human ARPE19 cells^22^ and rats^24^, the RPE-expressed CLIC4 in mice was also concentrated in the Lamp2-labeled late endocytic organelles (late endosome, lysosomes). The late endocytic organelles have emerging roles for modulating lipid metabolism, nutrient sensing, and membrane repair^56^. Furthermore, the RPE cells of the KO mice had increased the expression of autophagosomes. Human RPE cells isolated from AMD eyes also had a higher number of visible autophagosomes, but they were functionally impaired^57^. The attenuated expression of these organelles in the CLIC4 deficient RPE cells might affect some or all their functions; a subject of interest remains to be investigated.

Thus far, the etiology of drusen remains unclear. Drusen and atherosclerotic plaques are both lipid-rich extracellular deposits. In atherosclerotic plaques, ECM plays an important role in retaining lipids for modification (e.g., oxidation, aggregation, fusion)^58^. EM studies showed RPE^∆*Clic4*^ mouse BrMs had structurally altered ECM. The common DEGs (KO vs. Ctrl, CreCtrl, or WT) analyzed by DAVID revealed the enrichment of the ECM-receptor signaling pathway (Supplementary Fig. 6d). IPA revealed CLIC4 deficient RPE cells had suppressed matrix metallopeptidase (MMP) signaling (Fig. 4e). MMPs are a family of enzymes (e.g., MMP2, MMP14) that proteolytically degrade various ECM and surface proteins. *MMP2* is an AMD risk gene. RPE^∆*Clic4*^ mouse RPE cells expressed fewer *Mmp2* transcripts (Fig. 4b, Supplementary Fig. 6b). Our lab previously showed CLIC4-suppressed human ARPE19 cells had a reduced ability to degrade ECM due to impaired MMP14 and MMP2 secretion^22^. Thus, we propose that the attenuated gene expression, signaling, and secretion of the MMPs jointly contribute to the remodeling of BrMs juxtaposed to the CLIC4 deficient RPE cells.

A major advance in this paper is our demonstration of lipid-containing particles and spheres moving from the RPE into BrMs. This has not been shown conclusively in vivo before. The FIB-SEM studies revealed the previously unrecognized RPE export of LDs. The secreted LDs were smaller than the majority of RPE cytoplasmic LDs, indicating selectivity. Emerging evidence is showing that improperly metabolized lipids are expelled extracellularly^59^. Secreted LDs might have a direct link to the etiology of atherosclerotic lesions^60^. By analogy, we propose that the LDs released from the *Clic4*-KO RPE cells act as a seed in the sub-RPE/BrMs. The binding of LDs to lipoprotein may subject the latter to aggregation, BrM retention, and reduced choroidal clearance. Together, they serve as a platform to build future BLinDs. These results make sense because both the LDs and lipoproteins consist of a core that is rich in neutral lipids (cholesterol esters, triacylglycerol) and a surface rich in polar lipids (phospholipids, cholesterol)^60^. These components are also the main lipid species of drusen.

LDs have a dynamic shape and size, depending on the cell context. The LDs in the RPE have not been well characterized. Previous studies showed RPE cells express an LD-like organelle, the retinosome, which can sequester surplus all-*trans*-retinol or all-*trans*-retinyl esters^61,62^. The apical and subapical RPE staining of PLIN2 (Supplementary Fig. 7d) likely corresponds to the retinosomes^61^, though its ultrastructure is less well defined. The PLIN2 increased in apical RPE cells during normal aging was distinct from the LDs basally enriched in the KO mice.

*Clic4*-KO RPE cells had predicted PPAR/RXR/LXR pathway activation (Fig. 4e). Cholesterols (and their oxidized products oxysterols) are favorable ligands of the PPAR/RXR/LXR pathway. Like in other cell types, the lipid export role in RPE cells is probably carried out primarily by the cholesterol efflux pump ABCA1^63^. Lipid raft microdomains are the sites rich in ABCA1^64^ and export cholesterols^65,66^. Thus, the lipid raft loss in the basal plasma membranes of *Clic4*-KO RPE cells may impede cholesterol export. The lysosome misexpression-resulting lipid metabolism dysregulation may provide an additional mechanistic action explaining how *Clic4* deficiency might affect the lipid homeostasis of the RPE cells.

Finally, our demonstration that RPE cell-autonomous lipid dysregulation actively participates in drusen formation has significant clinical implications. It may help to explain the mixed clinical outcomes from treating AMD patients using statins (drugs that lower the cholesterol levels in the blood)^2^. We, however, cannot rule out the contribution of blood-derived lipids in drusen formation, particularly at the late-stage of the disease. In sum, the present study substantiates the model suggesting that lipid dysregulation predisposes to the genesis of drusen. Our mechanistic insights into drusen etiology open a door for developing new targets to treat AMD by reducing drusen.

## Methods

### Reagents

All antibodies and dyes used in this study and their validation are listed in Supplementary Table 2. All primers (Integrated DNA Technologies) used for genotyping and qPCR are listed in Supplementary Table 3. All chemicals were obtained from Sigma-Aldrich unless otherwise mentioned.

### Generation of RPE^∆*Clic4*^ mice

All procedures using mice were approved by the Weill Medical College of Cornell University Institutional Animal Care and Use Committee. Mice were maintained on a standard 12: 12-hour light: dark photoperiod in a vivarium maintained at 21.5 ± 1 °C, relative humidity of 30–70% and received unrestricted access to standard mouse chow (LabDiet 5053, PMI, St Louise, MO). Our *Clic4* ^f/f^ mice^16^ and *Best1*-Cre^+/−^ mice^27^ had the C57BL6/J background. We first generated *Best1-*Cre^+/−^; *Clic4*^f/WT^ mice and backcrossed these mice with *Clic4* ^f/f^ mice. We then bred *Best1-*Cre^+/−^; *Clic4* ^f/f^ males with CLIC4^f/f^ females to generate *Best1*-Cre^+/−^; *Clic4* ^f/f^ (RPE^∆*Clic4*^ or KO) and littermate control *Best1*-Cre^−/−^; *Clic4* ^f/f^ (Ctrl) mice. The RPE-specific Cre expression of *Best1*-Cre^+/−^ mice was reported to start postnatal 10–15 day-old^27^. Supplementary Table 1 lists the numbers, sexes, and genotypes of the mice used for each experiment.

### Fundus imaging and fundus lesion quantification

Mouse pupils were first dilated with 1% Tropicamide Ophthalmic Solution (Akorn) and 2.5% Phenylephrine Hydrochloride Ophthalmic Solution (Akorn) and then anesthetized by inhalation with isoflurane. After placing Refresh Lubricant Eye Drops (Allergan), the fundus and OCT scans were acquired using the Micron III-OCT2 system (Phoenix Research Labs). Infrared reflectance and autofluorescence scans were acquired using Spectralis HRA + OCT (Heidelberg Engineering, Heidelberg, Germany). A 55° angle lens was used, and projection images of 30 frames per fundus were taken, focusing on the outer retina.

To quantify the progression of the white-yellowish lesions, we used the Image Color Summarizer (http://mkweb.bcgsc.ca/color-summarizer/?). The percentage of the white lesion areas in the fundus image was determined by a color clustering function that partitioned the pixels into different color groups, which were then matched with the white color groups of the deposits in the fundus photographs.

### OMR analysis

For each mouse and condition, three independent trials were collected, on an OMR setup essentially as described^67^. For each trial, the stimulus was rotated at a constant speed but reversed in direction every 5 s, resulting in 12 stimulus epochs for a total of one minute. Stimuli were sinusoidal gratings rotating at 12 deg/sec, constant contrast = 1, over a range of spatial frequencies (0.025, 0.05, 0.1, 0.15, 0.2, 0.25, 0.3, 0.35, 0.4, 0.45, 0.5, 0.6) (Fig. 1f). Offline head tracking was performed using the OKRmonitor program^67,68^, and OMR indices are calculated as a ratio of frames in which the mouse head moved in the correct versus incorrect direction. For each mouse and each condition, three trials were performed, and median values of the three trials extracted (presented as individual data points in Fig. 1f, Supplementary Fig. 2). For visual acuity plots, curve fitting using the Matlab curve fitting toolbox (cftool) was performed, and optimal spatial frequency (with maximal OMRI) determined^67,68^. Statistical significance was calculated using the Kolmogorov-Smirnoff test (KS2) by Matlab 9.7.

### ERG and dark recovery analysis

ERG was performed using Espion e2 Visual Electrophysiology System (Diagnosys). After overnight dark-adaptation, mice were anesthetized with an intraperitoneal injection of mixture ketamine (100 mg/kg) and xylazine (20 mg/kg). The eyes of anesthetized mice were dilated with a drop of tropicamide and phenylephrine, and tetracaine (0.5%) was applied before ERG. Body temperature was maintained at 37 °C with a heating pad. A reference electrode was inserted subcutaneously between the eyes, and a ground electrode was inserted subcutaneously at the tail base and ERGs were recorded from both eyes using gold wire loops. Both eyes had the contact corneal electrodes held in place by a drop of Gonak solution (Akorn). ERG was performed using flashes with intensities ranging from 0.001 to 3 cd s/m^2^ for scotopic. For photopic recording, the stimulus was presented for 10 s with intensities from 0.3 to 100 cd s/m^2^. Mice were light adapted for 7 min before photopic recordings. Eight recordings were averaged per light intensity.

We used DC-ERG to record RPE-generated electrical responses by a 7 min 10 cd·s/m^2^ stimulus, as described^69^. The amplitude of the c-wave (i.e., the pre-stimulus baseline to the peak of the c-wave), the amplitudes of the fast oscillation (i.e., the c-wave peak to the trough of the fast oscillation), the light peak (i.e., fast oscillation trough to the asymptotic value), and the off-response amplitude (i.e., the light peak to the peak of the off response) were analyzed by AnalyzerDCERGv7.exe, as described^69^.

Dark recovery experiments were conducted as described^70^. Rod ERG a-wave maximal amplitude (Amax) was first determined in darkness at 23.5 cd s/m^2^ (average of 5 measurements). >90% of the relevant visual pigment was bleached with a 60 s exposure to 3900 cd sm^−2^ green light (~4.2 W·m^−2^; ~1.1 × 10^7^ photons μm^−2^ s^−1^) focused at the surface of the cornea. The bleached fraction was then estimated from the following equation: *F* = 1 – exp(−*I* × *P* × *t*), where *F* is the fraction of pigment bleached, *t* (second) is the duration of the light exposure, *I* is the bleaching light intensity of green light (~1.1 × 10^7^ photons μm^−2^ s^−1^), and *P* is the photosensitivity of mouse photoreceptors at the wavelength of peak absorbance (5.7 × 10^−9^ μm^2^ for mouse rods^71^). After bleaching, flash stimuli of 23.5 cd s/m^2^ were given every 5 min (five measurements were averaged) for a total of 90 min. In the beginning of the experiments, mice were anesthetized with an intraperitoneal injection of mixture ketamine (100 mg/kg) and xylazine (20 mg/kg). Every 30 min, mice were injected with ketamine (50 mg/kg) and xylazine (10 mg/kg), and Gonak: PBS (1:1) was reapplied to the cornea.

### Light microscopic histological, immunostaining, and lipid staining

For the histological staining of the retinal sections, the whole eye is placed in room temperature Excalibur’s Alcoholic Z-Fix (Excalibur Pathology) for a minimum of 48 h and then processed for paraffin embedding. 4 µm-thick sections of the entire globes were taken containing optic disc and pupil. The slides are stained with H&E and toluidine blue. We used the light microscopy acquired images of the H&E-stained paraffin sections to count the number of the pigmented cells localized at the subretinal spaces.

For the retinal immunostaining, eyecups were prepared from eyes enucleated from mice transcardially perfused with (1) 10 ml of heparin saline (20 units/ml), (2) 20 ml of 4% paraformaldehyde (PFA)/3.75% acrolein (Polysciences) in 0.1 M phosphate buffer (pH 7.4), (3) 60 ml of 4% PFA in 0.1 M phosphate buffer and followed by the overnight post-fixation with 4% PFA with 0.1% glutaraldehyde in 0.15 M cacodylate buffer (pH 7.4). Omitting acrolein in step (2) in some experiments did not affect the results. The eyecups were embedded in agarose and vibratome sectioned (40 μm-thick). The free-floating technique was used for antibody and dye staining.

Nile red (5 μg/ml in 75% glycerol)^72^ was incubated with the vibratome sections for 30 min. For Nile Red quantification, we acquired images using confocal microscopy, and counted the Nile Red-labeled sub-RPE granules in ~1.2 mm-long retinal sections per mouse. Oil Red O (3 mg/ml in 30% triethyl phosphate; filtered)^73^ was incubated with the prebleached vibratome sections for 60 min. For Oil Red O quantification, we acquired images using bright field light microscopy, and counted the Oil Red O-labeled BrM patches (>10 μm-long stretches of signal) in ~1.2 mm-long retinal sections per mouse. To prepare RPE whole mounts or flat mounts, we removed the anterior segments and retinas from the orientation-marked eyeballs and fixed them by 4% PFA for 1–4 h. All sections were stained using free-floating methods and imaged by confocal microscopy by a Zeiss LSM880.

### Transmission EM and FIB-SEM

For the EM experiments, all mice were harvested 2 h after light onset. Eyecups were prepared from the eyes enucleated from the mice transcardially perfused with (1) 10 ml of heparin saline (20 units/ml), (2) 20 ml of 4% paraformaldehyde (PFA)/3.75% acrolein (Polysciences) in 0.1 M phosphate buffer (pH 7.4), (3) 60 ml of 4% PFA in 0. 1 M phosphate buffer, and followed by (4) a post-fixed in 2.5% glutaraldehyde/0.1 M cacodylate buffer at 4 °C. About 120 µm-thick vibratome sections prepared from small pieces of eyecup (~2 × ~2 mm) were processed for *en bloc* fixation and staining, as described^74^. Briefly, after several washes in the cold cacodylate buffer containing 2 mM calcium chloride, specimens were incubated with 1.5% potassium ferrocyanide, 2 mM calcium chloride, and 2% osmium tetroxide in 0.15 M cacodylate buffer, pH 7.4 for 1 h on ice followed by treatment with the 10 mg/ml of thiocarbohydrazide solution for 20 min at room temperature and then with 2% osmium tetroxide fixation for 30 min at room temperature. The *en bloc*-stained tissues were dehydrated with graded ethanol and embedded in Epon. Ultrathin sections (72 nm) were collected on G400-Cu grids (Electron Microscopy Sciences) and were examined under a Philips CM10 microscope for conventional transmission EM analysis. For FIB-SEM, as described^74^, we subjected the en bloc-stained retinal tissue blocks to be precision milled every 20 nm (with a pixel resolution of ~5 nm/pixel in *x* and *y*). The block face images were collected by the FEI Helios NanoLab 650 microscope. Images were processed using Serial Sections Alignment Programs of IMOD/eTomo to correct drifting caused by the 30° angle from the block face during imaging.

### RNA isolation, RNAseq, bioinformatics analysis, and qRT-PCR of mouse RPE cells

Mouse RPE cells were purified using the previously described method^75^ with modifications. We prepared the eye cups (by removing the muscles and connective tissues from the enucleated eyes) in ice-cold HBSS-HEPES (10 mM) buffer. We then incubated the eyecups in 1.5 ml of HBSS-HEPES containing 1 mg/ml hyaluronidase for 45 min at 37 °C/5% CO_2_, followed by incubation in ice-cold HBSS-HEPES-Ca/Mg (1.26 mM CaCl_2_, 0.493 mM MgCl_2_, and 0.407 mM MgSO_4_) for 30 min on ice. We then removed the retinas and subjected the remaining RPE-choroid-sclera cups to incubation in 1.5 ml of 0.25% Trypsin EDTA (Thermo Fisher Scientific) for 45 min at 37 °C/5% CO_2_. Subsequently, we shook off the detached RPE cells in 1 ml of ice-cold HBSS-HEPES-Ca/Mg buffer. The RPE cells isolated from the two eyes of the same mouse were pooled and centrifuged (4 °C, 2000 rpm, 5 min). The total RNAs of the loosened mouse RPE cell pellets were isolated using the Absolutely RNA Nanoprep kit (Agilent). The total RNA integrity was checked using a 2100 Bioanalyzer (Agilent Technologies, Santa Clara, CA). RNA concentrations were measured using the NanoDrop system (Thermo Fisher Scientific). Preparation of RNA sample library and RNA-seq were performed by the Genomics Core Laboratory at Weill Cornell Medicine. Messenger RNA was prepared using the TruSeq Stranded mRNA Sample Library Preparation kit (Illumina, San Diego, CA), according to the manufacturer’s instructions. The normalized cDNA libraries were pooled and sequenced on Illumina NovaSeq6000 sequencer with pair-end 50 cycles.

Nucleic acid reads were aligned and mapped to the GRCm38 (mm10) mouse reference genome by STAR (Version2.5.2)^76^, and transcriptome reconstructions were performed by Cufflinks (Version 2.1.1) (http://cole-trapnell-lab.github.io/cufflinks/). The abundance of transcripts was measured with Cufflinks in Fragments Per Kilobase of exon model per Million mapped reads (FPKM)^77^. Gene expression profiles were constructed for differential expression, cluster, and principal component analyses with the DESeq2 package^78^. For differential expression analysis, comparisons between KO vs. WT, Ctrl, or CreCtrl groups were conducted using parametric tests where read-counts follow a negative binomial distribution with a gene-specific dispersion parameter. Corrected p-values were calculated based on the Benjamini-Hochberg method to adjust for multiple testing. Based on the statistics from the DESeq2 analysis, the volcano plot was made by using GraphPad Prism version 8 software. For GSEA^79,80^, to avoid using an arbitrary threshold to select genes, we used ranked gene lists rather than a threshold to call DEGs for pathway analysis. The three sets of the significant Hallmark pathways (FDR < 0.05) had 30 pathways in common. DAVID analysis (https://david.ncifcrf.gov/summary.jsp) was conducted using the common DEGs shared by all three sets (KO vs. Ctrl, CreCtrl, and WT). The canonical pathway enrichment analyses and upstream regulator pathway analyses were generated using Ingenuity Pathway Analysis (IPA; QIAGEN Bioinformatics). The parameters used for the cutoff of the significance of each pathway analysis were specified in the figure/table legends. The pathways shared by all three groups are presented.

For the heat maps, the mean and standard deviation of the FPKM values for each gene were calculated. The data were then normalized using the equation (FPKM value - Mean)/Standard Deviation. The heat maps were then created using Heatmapper (http://www1.heatmapper.ca/).

For real-time qPCR, first-strand cDNA was synthesized from total RNAs by using Oligo(dT)_20_ primer and SuperScript™ III First-Strand Synthesis System (Thermo Fisher Scientific). The PCR reactions were performed in a 20 μl reaction mixture by using *Power* SYBR™ Green PCR master mix (Applied Biosystems) and StepOnePlus Real-time PCR system (Applied Biosystems). Primers used were listed in Supplementary Table 3. Each reaction was performed in triplicate, and the cycle threshold (*Ct*) values of interesting genes were normalization to a housekeeping gene (*Gapdh*, *Hprt*). The genes’ relative expression levels are calculated by the 2(−∆∆Ct) method. The statistical analyses were performed using Student’s *t*-test.

### ELISA for 4-HNE assay

Mouse RPE/choroid cups were sonicated in RIPA buffer (50 mM Tri-Cl pH 7.4, 150 mM NaCl, 0.5% NP40, 0.5% sodium deoxycholate, 1 mM EGTA, 5% Glycerol) plus protease inhibitor/phenylmethylsulfonyl fluoride/phosphatase inhibitor). The homogenates were centrifuged for 15 min at 12,000 × *g* at 4 °C. Protein concentration was determined by BCA assay (Bio-Rad Laboratories) and 16 μg homogenates (in 50 μl) were used in each 96-well for 4-HNE concentration assay, as described^81^. 4-HNE concentration was quantified using an ELISA kit following the manufacturer’s protocol (#Ab238538, Abcam). The ELISA data analysis was collected by SPECTRA Max (450 nm) M2e plate reader (Molecular Devices Corp) and processed by SoftMax Pro 5.4. The average (presented as μg per ml) and SEM were calculated based on the 4-HNE-BSA standard curves.

### Statistics and reproducibility

The number of experimental replications and mice used are described in figure legends. Supplementary Table 1 summarizes the number and gender of the mice used for figure presentation. Statistical differences were determined using GraphPad Prism software version 6 or 8, or Microsoft Excel for Mac Version 16.16.27 (201012). The reported *P* values were obtained using a two-tailed Student’s *t*-test or two-tailed non-parametric Mann–Whitney *U*-test as described in figure legends. Differences between values were considered significant when *p  <  0.05, **p  <  0.01, ***p  <  0.001, ****p  <  0.0001.

### Reporting summary

Further information on research design is available in the Nature Research Reporting Summary linked to this article.

## Supplementary information

Supplementary Information

Description of Additional Supplementary Files

Supplementary Movie 1

Supplementary Movie 2

Supplementary Movie 3

Reporting Summary

## Data Availability

The raw Illumina RNA sequencing data generated in this study were deposited in the NCBI Sequence Read Archive (SRA) under the accession number GSE191077. The authors declare that data supporting the findings of this study are available within the main text and supplementary materials. The source data underlying Figs. 1a–f, 3k, 4a, 4b, and 5i and Supplementary Figs 2, 3b, 3d, 3e, 5c, 6a, 6b, 6c, 6f, 7g, 8c, and 9c are provided as a Source Data File. All data are available from the corresponding author (chsung@med.cornell.edu) upon reasonable request. Source data are provided with this paper.

## Source data

Source Data
