# Supplementary material for: Retinal pigment epithelium-specific CLIC4 mutant is a mouse model of dry age-related macular degeneration
Source: Nat Commun. 2022 Jan 18;13:374. doi: 10.1038/s41467-021-27935-9 (PMC8766482; doi:10.1038/s41467-021-27935-9)
Supplement: Supplementary file 1 — Supplementary Information [file 41467_2021_27935_MOESM1_ESM.pdf]

Supplementary Information for

Retinal pigment epithelium-specific CLIC4 mutant is a mouse model of dry age-related macular degeneration

Jen-Zen Chuang [1@#], Nan Yang[1@], Nobuyuki Nakajima[1@*], Wataru Otsu[1@**], Cheng Fu[1], Howard Hua Yang[2], Maxwell Ping Lee[2], Armaan Fazal Akbar[3], Tudor Constantin Badea[3, 4], Ziqi Guo[1], Afnan Nuruzzaman[1], Kuo-Shun Hsu[1$], Joshua L Dunaief [5], and Ching-Hwa Sung[1, 6#]

[1]Department of Ophthalmology, Weill Medical College of Cornell University, New York, USA; [2]The Laboratory of Cancer Biology and Genetics, Center for Cancer Research, National Cancer Institute, National Institutes of Health, Maryland, USA; [3] National Eye Institute, National institute of Health, Maryland, USA; [4] Research and Development Institute, Transilvania University of Brasov, School of Medicine, Brasov, Romania; [5]FM Kirby Center for Molecular Ophthalmology, Scheie Eye Institute, Department of Ophthalmology, Perelman School of Medicine, University of Pennsylvania, PA, USA; [6]Department of Cell and Developmental Biology, Weill Medical College of Cornell University, New York, USA.

[@] These authors contributed equally to this work

[#] Corresponding authors: Ching-Hwa Sung (chsung@med.cornell.edu) and Jen-Zen Chuang (jzchuang@med.cornell.edu).

Supplementary Table 1. Summary of N and gender of the experimental mice.

Assays	Age (months)	KO mice	Control mice			Presentation
		**KO** Best1-Cre+/-; Clic4f/f	**Ctrl** Clic4f/f	**CreCtrl** Best1-Cre+/-; Clic4WT/WT	**WT** C57BL6/J	
**Fundus**	3 ± 1	> 32 males	> 20 males	12 males 5 females		Figs. 2a-f, 2k, S3a, 3b, 3d, 3f, and 3g
	6 ± 1.5	> 18 males	> 3 males	> 6 males		
	9 ± 1	> 14 males	> 5 males	>3 males		
	12 ± 1.5	> 16 males	> 11 males	> 3 males		
	13.5-21	13 males	9 males 6 females	4 males		
**OCT**	3 ± 1	> 8 males	> 4 males	8 males 2 females		Figs. 2e, 2f, 2g, and 2k; S3f, and S3g.
	6 ± 1.5	> 13 males	> 6 males	> 6 males		
	9 ± 1	> 14 males	> 5 males	> 3 males		
	12 ± 1.5	> 16 males	> 11 males	> 4 males		
	> 19.5	> 7 males	> 11 males	> 3males		
**ERG**	1-2	6 males/12 eyes	3 males/6 eyes			Figs. 1a-d, S1
	3	8 males/16 eyes	8 males/16 eyes			Figs. 1a-d, S1
	6 ± 1	3 males/6 eyes	4 males/8 eyes			Figs. 1a-d, S1
	9 ± 1	7 males/14 eyes	6 males/12 eyes			Figs. 1a-d, S1
	12 ± 1	4 males/8 eyes	4 males/8 eyes			Figs. 1a-d, S1
**DC-ERG**	1	4 females / 8 eyes	2 males 3 females/10 eyes			Figs. S8b,8c
**Dark Recovery**	3	2 males,4 females/12 eyes	2 males, 4 females/12 eyes			Fig. 1e
	6	5 males/10 eyes	2 males,3 females/10 eyes			Fig. 1e
	9	4 males/8 eyes				

			1 male, 3 females/8 eyes			Fig. 1e
**OMR**	7 ± 1.5				1 male,3 females	Figs. 1f, S2
	9 ± 1.5	5 males, 2 females	4 females			Figs. 1f, S2
**Histology**	6-8	3 males	3 males	3 males	3 males	
	15-20	3 males	3 males	3 males		
**Retinal lipid staining**	3	>3 males				Figs. 5h, i
	5-9	>3 males	3 males	3 males		Figs. 5a. 5h, 5i, S7a
	12 ± 0.5	3 males	3 males	3 males		Figs. S7f, g
	17±1	3 males				Figs. 5 h, i
**RPE wholemounts staining**	6	>3 males	>3 males	3 males		Figs. 3j, k; S5c, S5d, S7b-d
	12 ± 0.5	>3 males	>3 males	> 3 males		Figs. 2l; S7b, c
**Retinal section immunostaining**	1-2	3 males	3 males	>3 males		Figs. S4a, b
	3	>3 males	>3 males			Figs. 3a, b, e, F; S9a
	6-9	>3 males	>3 males			Figs. 2j, 3g, 5b-d, g; S3c, S7e, S9b
	>12	>3 males	>3 males	>3 males		Figs. 5e-g
**qPCR**	3	9 males	3 males	3 males	6 males	Fig. 4a; S6a, S6B
**RNAseq**	3	7 males. 2 vs. Ctrl; 3 vs. WT 2 vs. CreCtrl;	2 males	2 males	3 males	Figs. 4c, S6c, S6f
**EM**	3 ± 1	1 male; 2 females	1 male	1 male	2 males; 1 female	Figs. 3d
	6 ± 3	2 males; 4 females	1 male	2 males		Figs. 3c, h, i. 6a-j; S5a, 5b, S8a. Video 1-3
	17-20	5 males	4 males			Figs. 6g-j, S8a
**4-HNE ELISA**	10-11	3 females	1 male, 2 females			Fig. S9c

Supplementary Table 2. List of the antibodies (Ab) and dyes. FFA: free fatty acid; TG: triglyceride; UC: unesterified cholesterol; EC: esterified cholesterol; PL: phospholipid; IgG: immunoglobulin.

Antibody or dye	Description	Host Organism	Manufacture	Cat# (clone)	Validation	Dilution
ApoE	1° Ab	Goat	Millipore	AB947	AB_2258475	1:500
ApoE	1° Ab	Goat	Santa Cruz	sc-6384	AB_634036	1:100
CD45	1° Ab	Rat	BD Biosciences	550539 (30F-11)	AB_2174426	1:50
CLIC4	1° Ab	Rabbit	Homemade	CUMC28 [1]	Chou et al.,[1]	1:100
Complement 3	1° Ab	Goat	MP Biomedicals	0855444	AB_2334469	1:50
Complement 3 (Activated), C3 cleaved fragments (C3b, iC3b, C3c)	1° Ab	Rat	Hycult Biotech	HM1065 (2/11)	AB_10130996	1:50
Cre	1° Ab	Mouse	Millipore	MAB3120 (2D8)	AB_2085748	1:500
Ezrin	1° Ab	Mouse	Abcam	ab4069 (3C12)	AB_304261	1:200
Iba1	1° Ab	Rabbit	Wako	019-19741	AB_839504	1:500
Lamp2	1° Ab	Rat	DSHB	GL2A7 (P3U1)	AB_2314734	1:10
MCT3	1° Ab	Rabbit	Nancy J Philp		Philp et al.,[2]	1:5000
PLIN2	1° Ab	Rabbit	Proteintech	15294-1-AP	KO validated	1:500
PLVAP	1° Ab	Rat	DSHB	MECA-32 (SP2/0)	AB_531797	1:200
Vimentin	1° Ab	Mouse	Amersham	RPN1102	Sahlgren et al., [3]	1:30
Vimentin	1° Ab	Mouse	DAKO	M0725 (V9)	AB_10013485	1:500
Vitronectin	1° Ab	Mouse	Santa Cruz	sc-74484 (D-8)	AB_1131298	1:100
Alexa488 conjugated anti-goat IgG	2° Ab	Donkey	Thermo Fisher	A11055	AB_2534102	1:400
Alexa568 conjugated anti-goat IgG	2° Ab	Donkey	Thermo Fisher	A11057	AB_2534104	1:400
Alexa568 conjugated anti-mouse IgG	2° Ab	Donkey	Thermo Fisher	A10037	AB_2534013	1:400
Alexa488 conjugated anti-rabbit IgG	2° Ab	Donkey	Thermo Fisher	A21206	AB_2535792	1:400
Alexa568 conjugated anti-rabbit IgG	2° Ab	Donkey	Thermo Fisher	A10042	AB_2534017	1:400
Alexa594 conjugated anti-rat IgG	2° Ab	Donkey	Jackson	712-585 -153	AB_2340689	1:400

Alexa 488 conjugated Phalloidin dye	F-actin dye		Thermo Fisher	A12379	AB_2315147	1:400
Alexa 647 conjugated Phalloidin	F-actin dye		Thermo Fisher	A22287	AB_2620155	1:400
DAPI	Nuclear dye		Sigma	D9542	NA	0.3 μM
Nile Red	Lipid (FFA, TG, UC, EC, PL) dye		Sigma	19123	NA	5mg/ml
Oil Red O	Lipid (FFA, TG, EC) dye		Sigma	O0625	NA	3mg/ml

Supplementary Table 3. List of the primers.

Gene ID		Primer	Assay used
Clic4	Forward	5'-CATGTGCCACCACCACCAGA-3'	Genotyping (WT, 219 bp; Floxed, 259 bp)
	Reverse	5'-GACCAAGCTGGCCTCCAATTA-3'	
Best1-Cre #1	Forward	5'-AGGTGTAGAGAAGGCACTTAGC-3'	Genotyping (*Best1*-Cre, 477 bp)
	Reverse	5'-CTAATCGCCATCTTCCAGCAGG-3'	
Best1-Cre #2	Forward	5'-ATGCCCAAGAAGAAGAGGAAGGTGTCC-3'	Genotyping (*Best1*-Cre, 320 bp)
	Reverse	5'-TGGCCCAAATGTTGCTGGATAGTTTTTA-3'	
Crb1	Forward1	5'-GTGAAGACAGCTACAGTTCTGATC-3'	Genotyping (WT, 220 bp; ; *rd8*; 244bp)
	Forward2	5'-GCCCCTGTTTGCATGGAGGAAACTT GGAAGACAGCTACAGTTCTTCTG-3'	
	Reverse	5'-GCCCCATTTGCACACTGATGAC-3'	
Clic4	Forward	5'-TGGTGAAAGCATTGGAAACT-3'	qPCR
	Reverse	5'-GGCACAAGACTTCTTCGAGA-3'	
Dcn	Forward	5'-ACATAACTGCGATCCCTCAAG-3'	qPCR
	Reverse	5'-ACTGCCATTCTCCATAACGG-3'	
Gapdh	Forward	5'-CCTTCCGTGTTCCTACCC-3'	qPCR
	Reverse	5'-CAACCTGGTCCTCAGTGTAG-3'	
Hprt	Forward	5'- TCAGTCAACGGGGGACATAAA-3'	qPCR
	Reverse	5'-GGGGCTGTACTGCTTAACCAG-3'	
Ler3	Forward	5'-GCGCGTTTGAACACTTCTC-3'	qPCR
	Reverse	5'-ATGGCGAACAGGAGAAAGAG-3'	
Mmp2	Forward	5'-CATGATCAACTTTGGACGCTG-3'	qPCR
	Reverse	5'-ACTTGCAGTACTCGCCATC-3'	
Plvap	Forward	5'-AACTGTTGACTACGCGACG-3'	qPCR
	Reverse	5'-CGGCGATGAAGCGATTATAGT-3'	
Rho	Forward	5'-CCCTTCTCCAACGTCACAGG-3'	qPCR
	Reverse	5'-TGAGGAAGTTGATGGGGAAGC-3'	
Rpe65	Forward	5'-ACCACTAACAGCTCATGTCAC-3'	qPCR
	Reverse	5'-TGATAGAAAGGCTCAGATCCAAC-3'	

Supplementary Figures

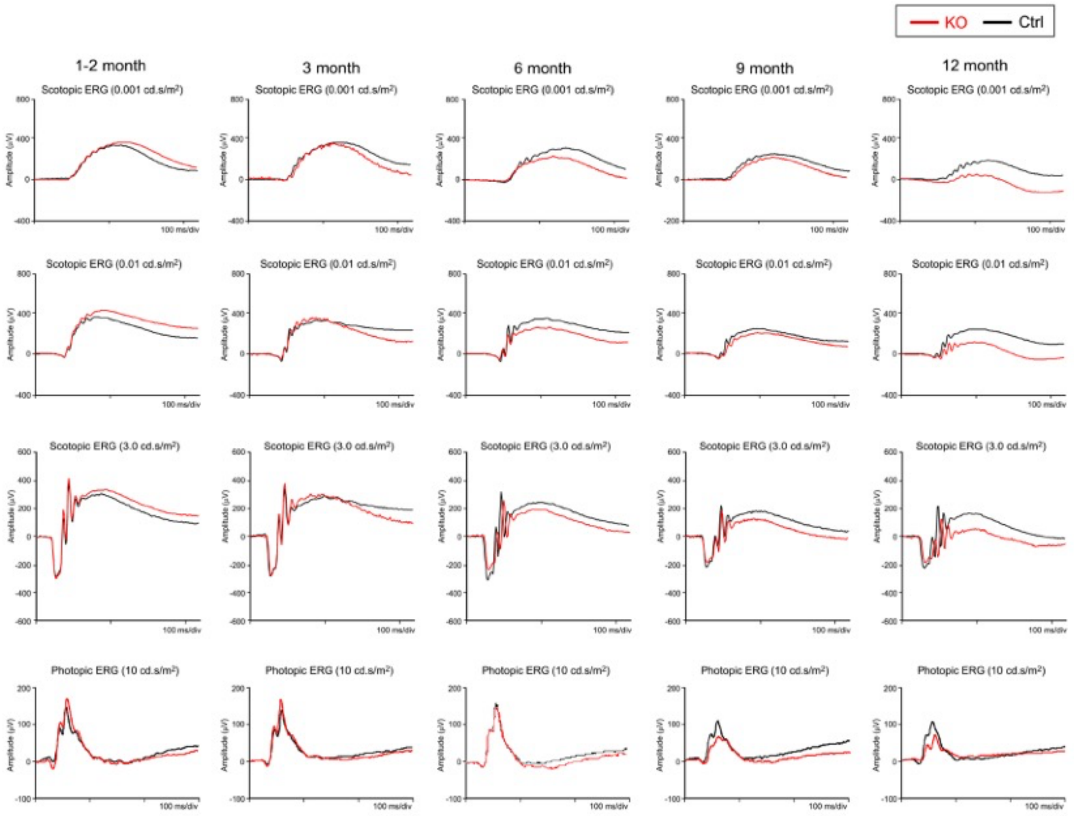

Supplementary Figure 1. RPE$^{\Delta Clic4}$ mice developed age-related ERG signal decline. Related to Fig. 1a-d.

Representative scotopic (top three rows; 0.001, 0.01, and 3.0 cd.s/m^2) or photopic (bottom row; 10 cd.s/m^2) traces of ERG responses of Fig. 1a-d are shown for indicated ages of Ctrl (black trace) and KO (red trace) mice.

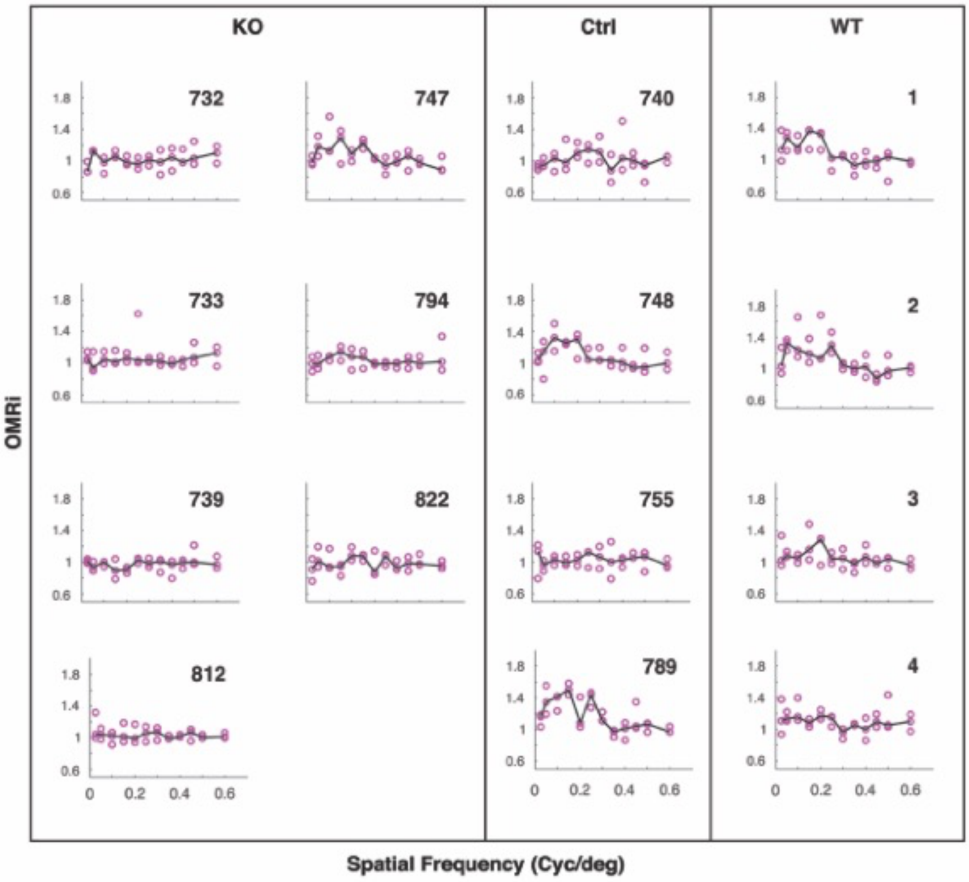

Supplementary Figure 2. Spatial frequency curve comparison between KO, Ctrl, and WT mice. Related to Fig. 1f.
Spatial frequency curves of the individual mice used for data presented in Fig. 1f. Dots represent OMRi for three independent trials per mouse. The individual mouse IDs are listed. Black lines are medians. Source data are provided as a Source Data file.

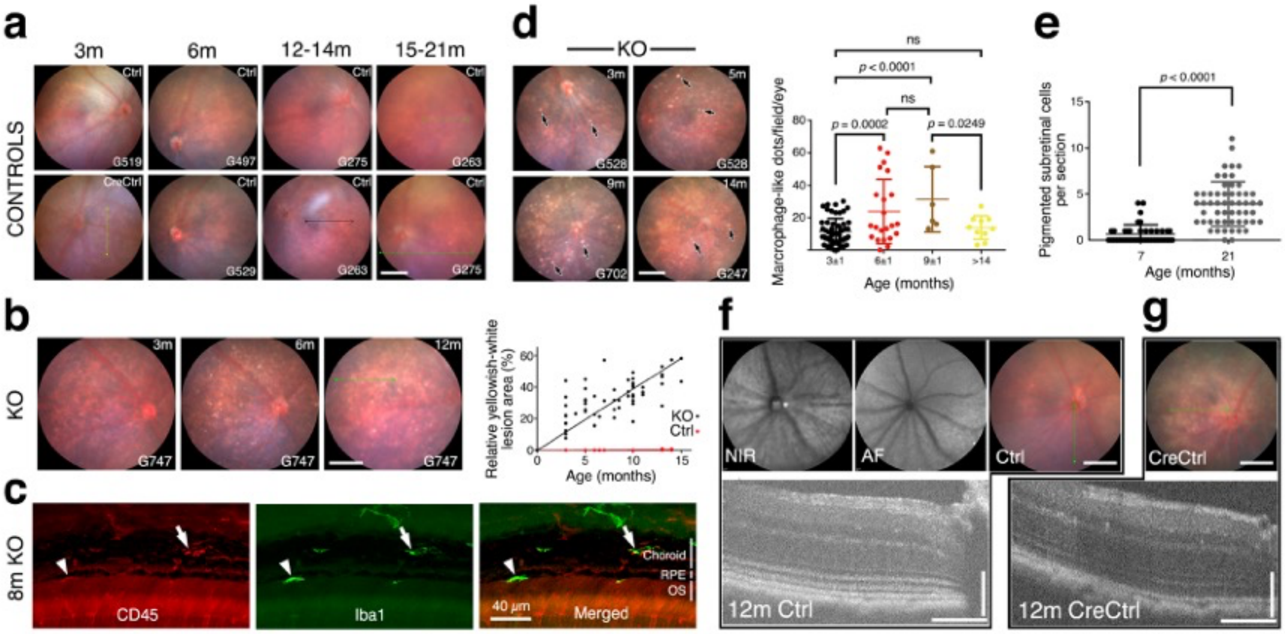

Supplementary Figure 3. Age-related fundus and retinal histopathological changes of RPE$^{\Delta Clic4}$ mice. Related to Figure 2.
(**a**) shows the absence of fundus lesions in a gallery of 3-to-21-month-old mice of Ctr and CreCtrl mice. Representative images of more than 3 independent experiments are shown; also see Supplementary Table 1.
(**b**) shows the fundus changes in a single mouse over time (left) and quantification of the yellowish-white fundus lesions (right). Each dot represents one mouse eye. n=11, 15, 20, 11, and 3 for 3±1, 6±1, 9±1, 12±1, and 14±1-month-old mice, respectively.
(**c**) shows an Iba1^{+}/CD45^{+} macrophage in the subretinal space (arrowhead) and choroid (arrow) in an 8-month-old KO mouse. Representative images of 3 experiments are shown.
(**d**) shows macrophage (and/or microglia)-like bright circular lesions in representative KO mouse fundi (left) and their quantification (right). Each dot represents the lesion number counted in one fundus image of a single eye. Data collected from 56, 22, 6, and 10 eyes of 3±1, 6±1, 9±1, and >14-month-old mice, respectively are shown in Mean ± SD. Two-tailed Student's *t*-test.
(**e**) shows the number of the subretinally localized pigmented cell bodies in 7-month-old (42 surveyed areas in N=3 mice) and 21-month-old (55 surveyed areas in N=3 mice) KO mice detected in the ~3.4mm long, 4μm-thick retinal sections. Mean ± SD and P-values (two-tailed Student's t-test) are shown. Of note, based on Toluidine blue staining, a small fraction of the pigmented cells might represent the pigment-engulfed immune cells in the 7-month-old mice. The Toluidine blue labeled immune cells were rarely seen in the aged mice, in agreement with the data in Fig. S3d.
(**f**) shows the near infrared (NIR), autofluorescence (AF), color fundus, and OCT of a 12-month-old Ctrl mouse. Representative images of 3 experiments are shown.
(**g**) shows the color fundus and SD-OCT of a 12-month-old CreCtrl mouse. Representative images of 3 experiments are shown.
The individual mouse IDs are shown in the fundus images (a, b, d, f). Scale bar = 400 (fundus photographs in a, b, d, f, g), and 100 (OCT images in f, g) μm. Source data of (b, d, e) are provided as a Source Data file.

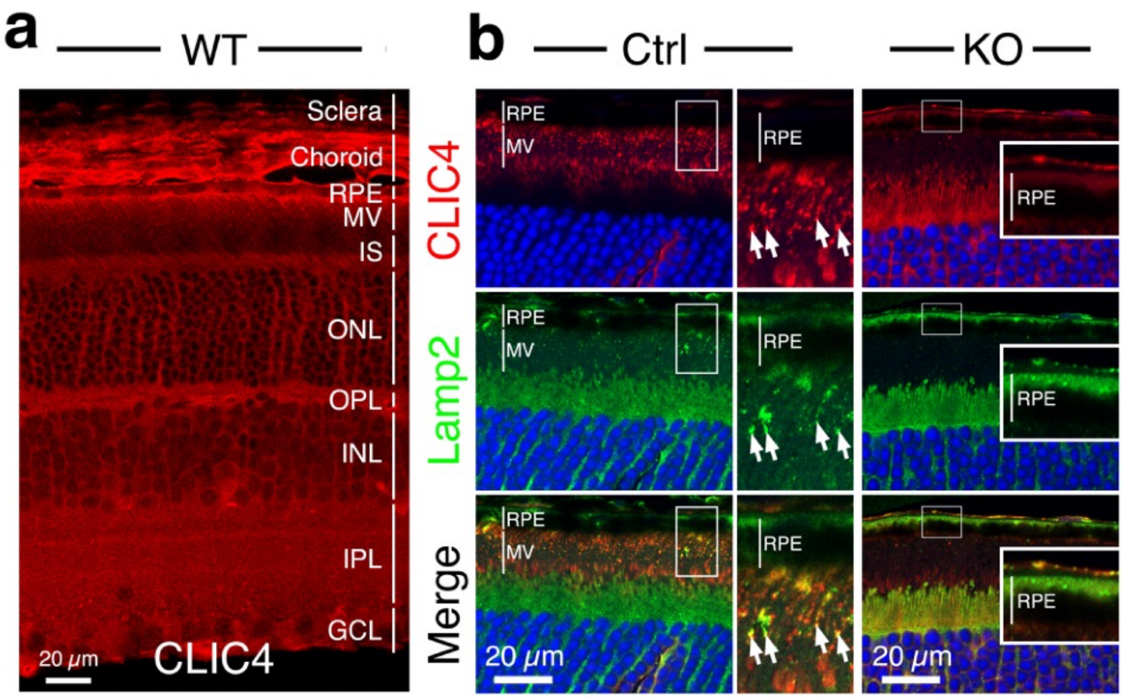

Supplementary Figure 4. CLIC4 expression in the retina-RPE-choroid complex. Related to Figure 3.
(**a**) 1-month-old WT mouse retinal section stained for CLIC4 (red). IS: inner segment; ONL: outer nuclear layer; INL; inner nuclear layer; OPL: outer plexiform layer; IPL: inner plexiform layer; GCL: ganglion cell layer. Representative images of at least 3 experiments are shown.
(**b**) 2-month-old mice (N=3) show CLIC4- and Lamp2-labeled puncta overlap in the RPE MVs of Ctrl mice (arrows). The 2-month-old KO mice had reduced RPE signals from CLIC4 and Lamp2. The remainder of Lamp2 was detected at the basal side of RPE monolayers. Representative images of 3 experiments are shown.

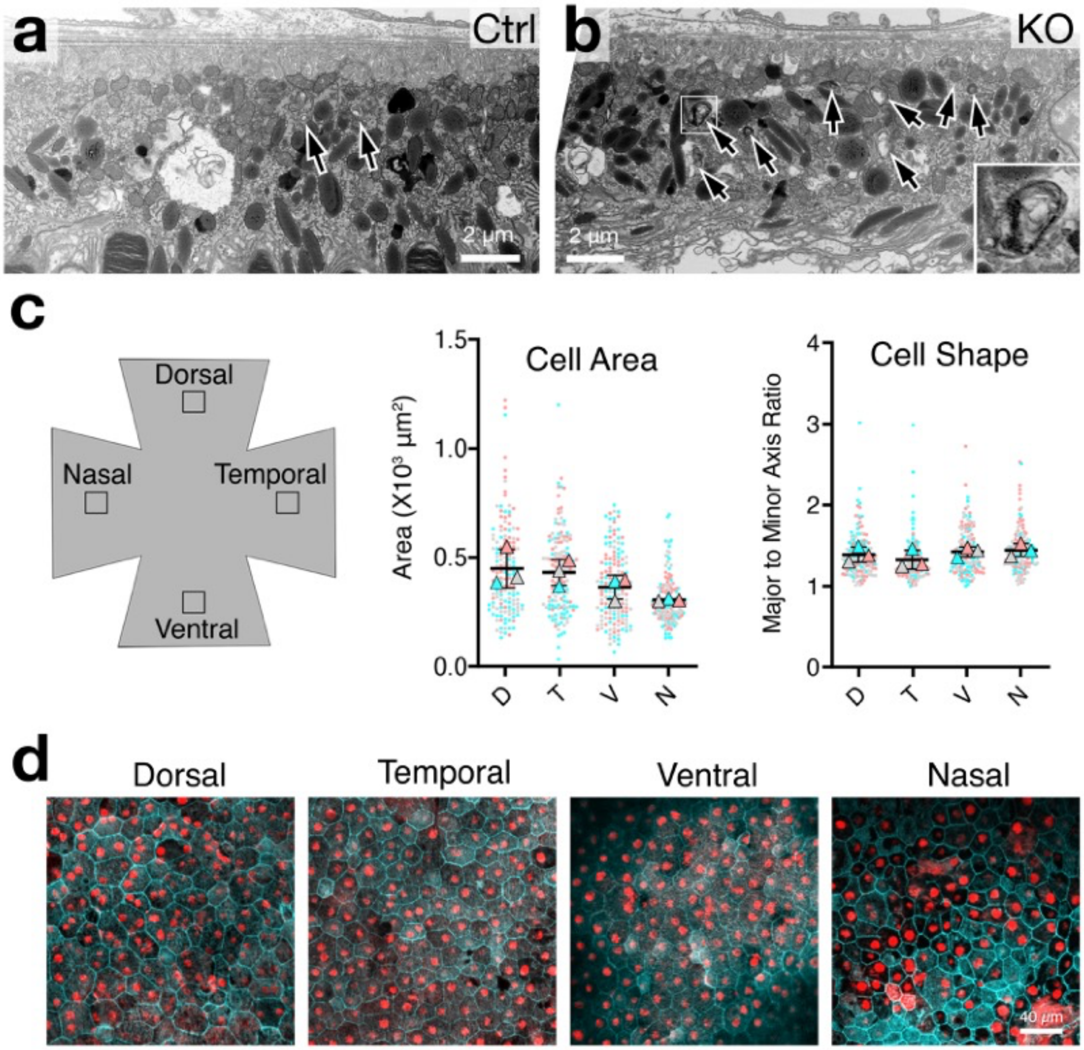

Supplementary Figure 5. Autophagosome, cell size/shape, and Cre expression of RPE$^{\Delta Clic4}$ mice. Related to Fig. 3.

(**a, b**) Compared to the 6-month-old Ctrl mice (a), the age-matched KO (b) mice showed markedly more autophagosomes (arrows) at the basal side of the RPE cells. The autophagosomes often contained multilamellar dark membranes (inset: enlarged boxed area). Representative images of 3 experiments are shown. (**c**) Left: A schematic diagram depicting the survey areas of 6-month-old RPE flat mounts for F-actin staining. Right: Mean ± SD of the cell area and cell shape of 6-month-old KO RPE cells in 4 different surveyed areas (D: dorsal; T: temporal; V: ventral; N: nasal) of N=3 mouse eyes (50 cells/area) are shown. Not significant by Two-tailed Student *t*-test.

(**d**) *En face* views of F-actin (cyan) and Cre recombinase (red)-labeled RPE sheets of different topographic regions in 3-month-old KO mice. Heterogenous cell shapes are readily observed. Representative images of 3 experiments are shown.

Source data of (c) are provided as a Source Data file.

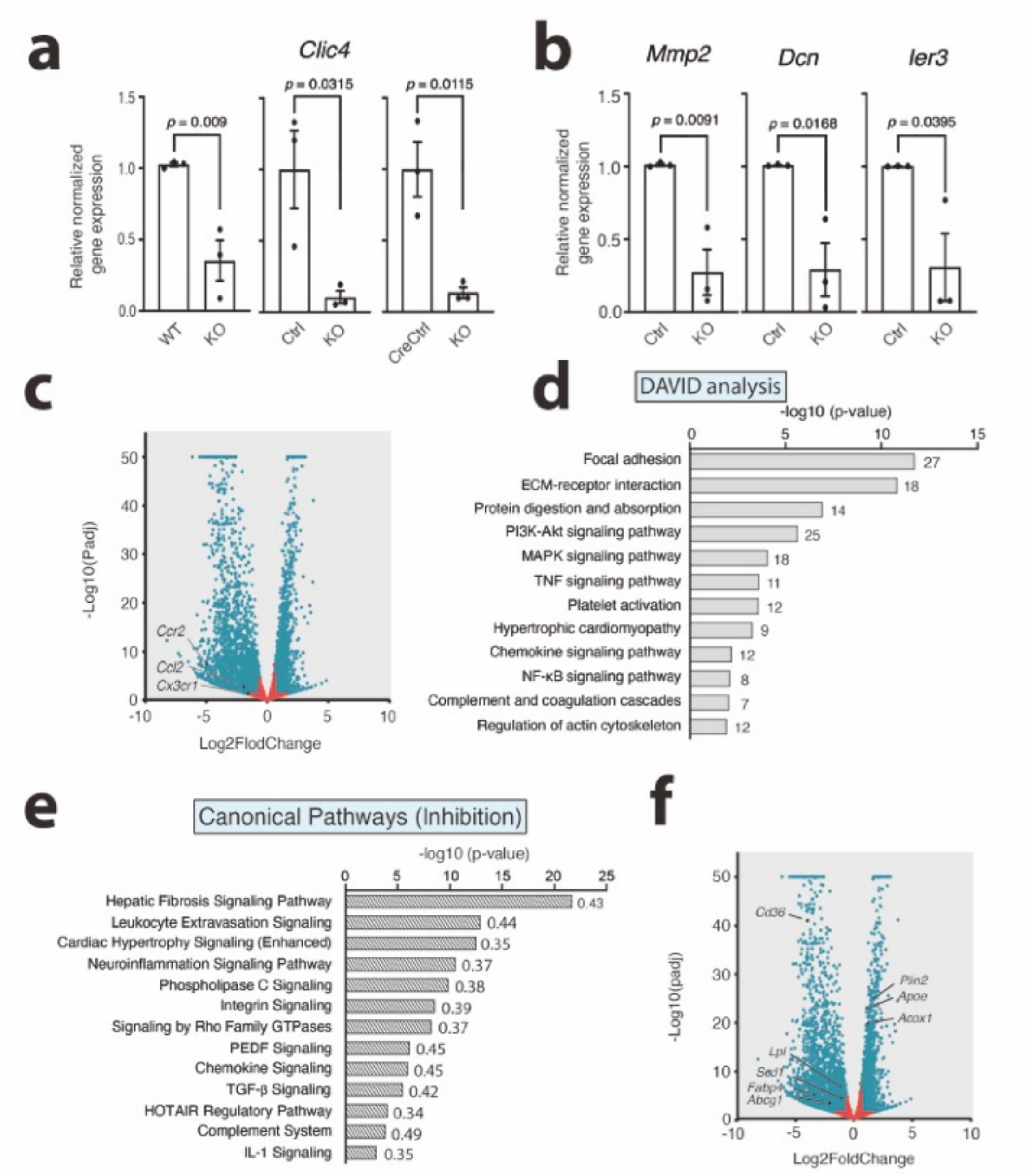

Supplementary Figure 6. Gene expression and DEG-based pathway analyses of CLIC4 deficient RPE cells. Related to Fig. 4.

(**a**) qPCRs demonstrate RPE loss of CLIC4 in 3-month-old KO (vs. WT, Ctrl, and CreCtrl) mice. Data are shown as means ± SEM (N=3). Two-tailed Student's t-test.

(**b**) Supporting evidence of RNAseq. qPCR results of the indicated AMD-risk genes using RNAs isolated from RPE cells of 3-month-old Ctrl and KO mice. Data are shown as means ± SEM (N=3). Two-tailed Student's t-test.

(**c**) Volcano plot (KO vs. WT RPE cells) with the AMD-related immunoregulatory genes marked.

(**d**) Examples of the DAVID analyses identified KEGG pathways that were enriched by the common DEGs shared by three groups (KO vs. WT, Ctrl, and CreCtr RPE cells). The P-values and the numbers of the affected genes are shown.

(**e**) Examples of the IPA analysis identifying canonical pathways (inhibition) common in all three groups. The P-values and the ratios from the KO vs. WT group are shown.
(**f**) Volcano plot of the DEGs (KO vs. WT RPE cells). Several PPAR/LXR/RXR targets are marked.
For (c) and (f), the DEG that had -log10($_{Padj}$) > 50 were truncated as 50 in the figure. Padj<0.05; FC>1.5 were considered as significantly changed (Cyan). Red indicates genes that were not significantly changed. Source data of (a, b, c, f) are provided as a Source Data file.

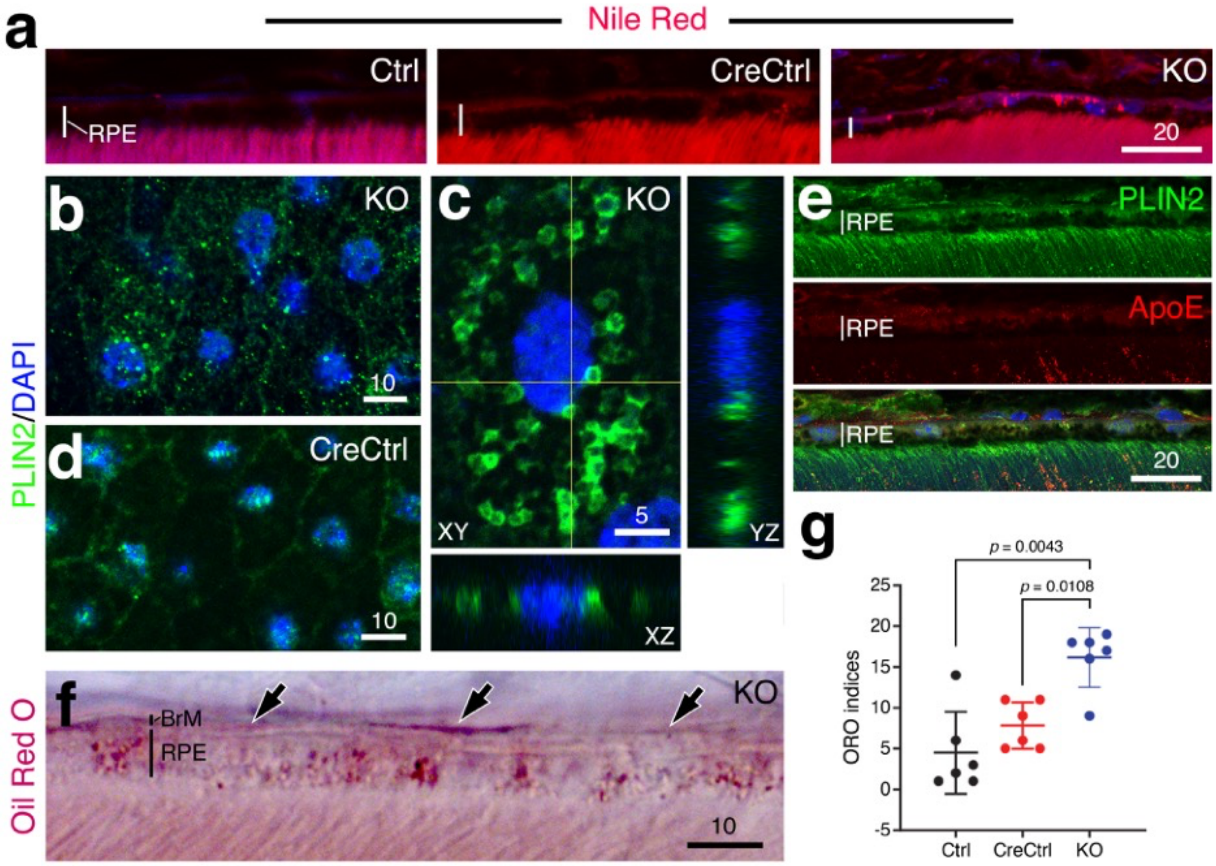

Supplementary Figure 7. Dysregulated lipid and lipoprotein expression of RPE$^{\Delta Clic4}$ mice
Related to Fig. 5.
(**a**) Bright Nile Red stained large RPE cytoplasmic LDs and BrM of 6-month-old KO, but not age-matched Ctrl and CreCtrl mice.
(b-d) PLIN2 (and DAPI) staining of RPE flat mounts of KO (b, c; 12 ± 0.5-month-old; N=3) and CreCtrl (d; 3-month-old, N=3) mice. Confocal images sectioned through the middle-basal (b, c) and apical (d) regions of KO and Cre-Ctrl RPE cells, respectively are shown. Enlarged views in (**c**) highlight the ring-shape, LD surface staining of PLIN2.
(**e**) 6-month-old CreCtrl mouse retinal section stained for ApoE and PLIN2.
Representative images of n=3 biologically independent samples are shown in (a-e).
(**f)** shows a representative image of bright Oil Red O-stained BrM patches in12-18-month-old KO mice (arrows).
(**g**) Quantification of the Oil Red O (ORO)-stained BrM-localized deposits (indices; see Methods) in 12-montho-old Ctrl, CreCtr, and KO mice. Mean ± SD of 6 surveyed areas in N=3 independent samples are shown. Two-tailed non-parametric Mann-Whitney U-test.
Scale bars (a-f) are shown in μm. Source data of (g) are provided as a Source Data file.

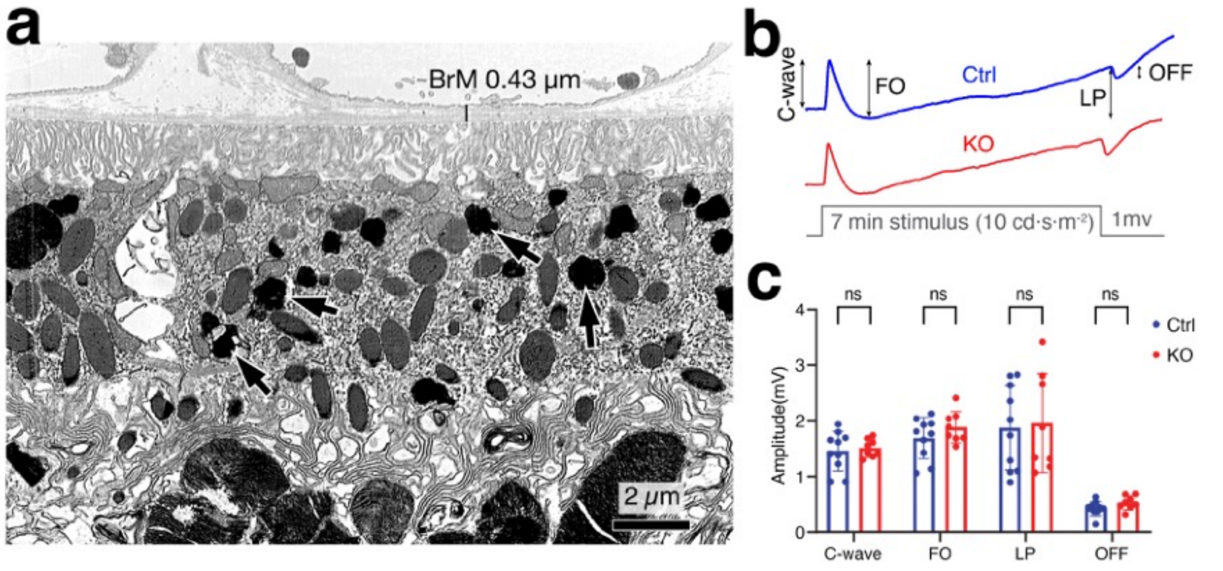

Supplementary Figure 8. TEM of aged Ctrl RPE (a) and DC-ERG of young mice (b, c). Related to Fig. 6 and Discussion.
(**a**) TEM image of the RPE-BrM-choroid complex of 18-month-old Ctrl mice (N=3). The notable senescence effects include increased lipofuscins (arrows) and less organized MVs.
(**b, c**) Representative DC-ERG tracing for 1-month-old Ctrl and KO mice recorded in response to a 7-min light stimulus. Amplitude (Mean ± SD) of the major components (FO: fast oscillation; LP: light peak; OF: off-response) of the DC-ERG from N=10 eyes (Ctrl) and N=8 eyes (KO) are shown. p = 0.695, 0234, 0.831 and 0.098 for c-wave, FO, LP and OFF, respectively. Not significant (ns). Two-tailed Student's t-test. Source data of (c) are provided as a Source Data file.

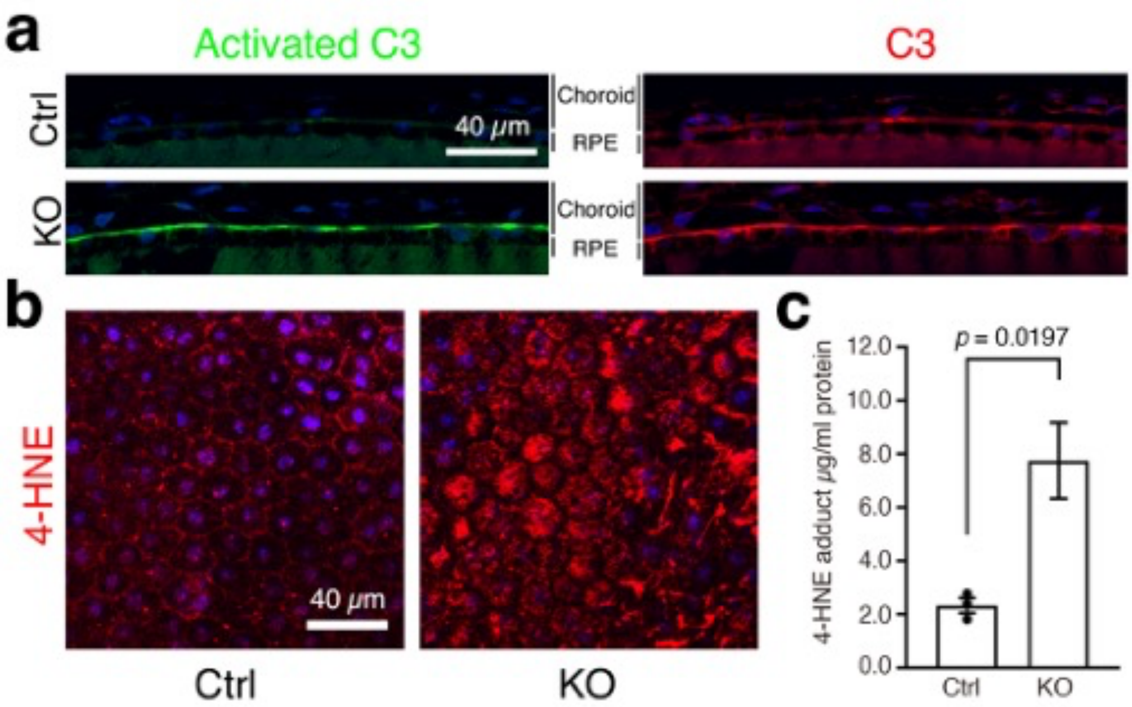

Supplementary Figure 9. Complement activation and oxidative stress of CLIC4 deficient RPE cells. Related to Discussion.
(**a**) RPE-choroid region of 3-month-old Ctrl and KO mice co-labeled with activated C3 and C3 cleaved products C3b/iC3b/C3c (green) as well as C3 (red). Blue: DAPI nuclear staining. Representative images of 3 independent experiments are shown.
(**b**) 4-HNE (red) and DAPI (blue) staining of 6-month-old Ctrl and KO mouse RPE flat mounts.
(c) ELISA quantification of 4-HNE in 10-month-old Ctrl and KO mice. Mean ± SEM (N=3) are shown. Two-tailed Student's *t*-test. S Source data of (c) are provided as a Source Data file.

Supplementary References

1 Chou, S. Y. *et al.* CLIC4 regulates apical exocytosis and renal tube luminogenesis through retromer- and actin-mediated endocytic trafficking. *Nat Commun* **7**, 10412, doi:10.1038/ncomms10412 (2016).

2 Philp, N. J., Yoon, H. & Grollman, E. F. Monocarboxylate transporter MCT1 is located in the apical membrane and MCT3 in the basal membrane of rat RPE. *Am J Physiol* **274**, R1824-1828 (1998).

3 Sahlgren, C. M. *et al.* Mitotic reorganization of the intermediate filament protein nestin involves phosphorylation by cdc2 kinase. *J Biol Chem* **276**, 16456-16463, doi:10.1074/jbc.M009669200 (2001).
