# Supplementary material for: Retinal pigment epithelium-specific CLIC4 mutant is a mouse model of dry age-related macular degeneration
Source: Nat Commun. 2022 Jan 18;13:374. doi: 10.1038/s41467-021-27935-9 (PMC8766482; doi:10.1038/s41467-021-27935-9)
Supplement: Supplementary file 2 — Description of Additional Supplementary Files [file 41467_2021_27935_MOESM2_ESM.pdf]

Description of Additional Supplementary Files

File Name: Supplementary Movie 1

Description: EM tomography related to Fig. 6. Stacked FIB-SEM images (covering 400 nm of z-thickness) depict the RPE-BrM-choroid complex of a 6-month-old CreCtrl mouse. Granular-shaped lipoprotein-like particles (arrows) emerge from the dark lipid raft microdomains expressed in the “endfoot” of the basal infoldings (arrowheads). The BrM displays plenty of similar lipoprotein-like particles (arrowheads). Fig. 6a is a single frame from this series of images.

File Name: Supplementary Movie 2

Description: EM tomography related to Fig. 6. Stacked FIB-SEM images (covering 200 nm of z-thickness) depict the RPE-BrM-choroid complex of a 6-month-old KO mouse. Open arrows point to an LD that emerges from the RPE cytosol to the subRPE extracellular space (yellow arrows). Fig. 6c and Supplementary Fig. 5 show two and six frames of this video, respectively.

File Name: Supplementary Movie 3

Description: EM tomography related to Fig. 6. Stacked FIB-SEM images of a 6-month-old KO mouse RPE-BrM-choroid complex (covering 560 nm of z-thickness). Arrows mark the LDs in the subRPE spaces that enter the BrM near the RPE basement membranes (BM).
