# Supplementary material for: Retinal pigment epithelium-specific CLIC4 mutant is a mouse model of dry age-related macular degeneration
Source: Nat Commun. 2022 Jan 18;13:374. doi: 10.1038/s41467-021-27935-9 (PMC8766482; doi:10.1038/s41467-021-27935-9)
Supplement: Supplementary file 6 — Reporting Summary [file 41467_2021_27935_MOESM6_ESM.pdf]

nature portfolio

Corresponding author(s): Ching-Hwa Sung

Last updated by author(s): October 19, 2021

Reporting Summary

Nature Portfolio wishes to improve the reproducibility of the work that we publish. This form provides structure for consistency and transparency in reporting. For further information on Nature Portfolio policies, see our Editorial Policies and the Editorial Policy Checklist.

Statistics

For all statistical analyses, confirm that the following items are present in the figure legend, table legend, main text, or Methods section.

n/a	Confirmed	
☐	☒	The exact sample size (*n*) for each experimental group/condition, given as a discrete number and unit of measurement
☐	☒	A statement on whether measurements were taken from distinct samples or whether the same sample was measured repeatedly
☐	☒	The statistical test(s) used AND whether they are one- or two-sided<br>*Only common tests should be described solely by name; describe more complex techniques in the Methods section.*
☒	☐	A description of all covariates tested
☒	☐	A description of any assumptions or corrections, such as tests of normality and adjustment for multiple comparisons
☐	☒	A full description of the statistical parameters including central tendency (e.g. means) or other basic estimates (e.g. regression coefficient) AND variation (e.g. standard deviation) or associated estimates of uncertainty (e.g. confidence intervals)
☐	☒	For null hypothesis testing, the test statistic (e.g. *F*, *t*, *r*) with confidence intervals, effect sizes, degrees of freedom and *P* value noted<br>*Give P values as exact values whenever suitable.*
☒	☐	For Bayesian analysis, information on the choice of priors and Markov chain Monte Carlo settings
☒	☐	For hierarchical and complex designs, identification of the appropriate level for tests and full reporting of outcomes
☒	☐	Estimates of effect sizes (e.g. Cohen's *d*, Pearson's *r*), indicating how they were calculated

Our web collection on statistics for biologists contains articles on many of the points above.

Software and code

Policy information about availability of computer code

Data collection	Commercial software used include http://mkweb.bcgsc.ca/color-summarizer/?; STAR (Version2.5.2); Cufflinks (Version 2.1.1) (http://cole-trapnell-lab.github.io/cufflinks/);DESeq2 package ; GraphPad Prism version 6; GraphPad Prism version 8 ; https://david.ncifcrf.gov/summary.jsp; Heatmapper (http://www1.heatmapper.ca/; SoftMax Pro 5.4. Open access software used include OKRmonitor program and OMR arena (published in doi:10.1016/j.jneumeth.2015.08.007 (2015); doi:10.1371/journal.pone.0078058 (2013); contacting author Dr. Tudor Badea); AnalyzerDCERGv7.exe (published in doi:10.3791/61491 (2020; contacting authors Dr. Wei Li and Kiyoharu Miyagishima.
Data analysis	For OMR analysis, offline head tracking was performed using the OKRmonitor program. OMR indices are calculated as a ratio of frames in which the mouse head moved in the correct versus incorrect direction. For visual acuity plots, curve fitting using the Matlab curve fitting toolbox (cftool) was performed, and optimal spatial frequency (with maximal OMRI) determined 3,4. Statistical significance was calculated using the Kolmogorov-Smirnoff test (KS2) under Matlab (version 9.7).<br>For DC-ERG, we used the published software AnalyzerDCERGv7.exe for analyzing the amplitude of the c-wave (i.e., the pre-stimulus baseline to the peak of the c-wave), the amplitudes of the fast oscillation (i.e., the c-wave peak to the trough of the fast oscillation), the light peak (i.e., fast oscillation trough to the asymptotic value), and the off-response amplitude (i.e., the light peak to the peak of the off response).<br>To quantify the progression of the white-yellowish lesions, we used the Image Color Summarizer (http://mkweb.bcgsc.ca/color-summarizer/?).<br><br>For RNAseq data analysis, nucleic acid reads were aligned and mapped to the GRCm38 (mm10) mouse reference genome by STAR (Version2.5.2)12, and transcriptome reconstructions were performed by Cufflinks (Version 2.1.1) (http://cole-trapnell-lab.github.io/cufflinks/). The abundance of transcripts was measured with Cufflinks in Fragments Per Kilobase of exon model per Million mapped reads (FPKM) 13. Gene expression profiles were constructed for differential expression, cluster, and principal component analyses with the DESeq2 package 14. For differential expression analysis, comparisons between KO vs. WT, Ctrl, or CreCtrl groups were conducted using parametric tests where read-counts follow a negative binomial distribution with a gene-specific dispersion parameter. Corrected p-values were calculated based on

the Benjamini-Hochberg method to adjust for multiple testing. Based on the statistics from the DESeq2 analysis, the volcano plot was made by using GraphPad Prism version 8 software. For GSEA 15,16, to avoid using an arbitrary threshold to select genes, we used ranked gene lists rather than a threshold to call DEGs for pathway analysis. The three sets of the significant Hallmark pathways (FDR<0.05) had 30 pathways in common. DAVID analysis (https://david.ncifcrf.gov/summary.jsp) was conducted using the common DEGs shared by all three sets (KO vs. Ctrl, CreCtrl, and WT). The canonical pathway enrichment analyses and upstream regulator pathway analyses were generated using Ingenuity Pathway Analysis (IPA; QIAGEN Bioinformatics). The parameters used for the cutoff of the significance of each pathway analysis were specified in the figure/table legends. The pathways shared by all three groups are presented.

For the heat maps, the mean and standard deviation of the FPKM values for each gene were calculated. The data were then normalized using the equation (FPKM value - Mean)/Standard Deviation. The heat maps were then created using Heatmapper (http://www1.heatmapper.ca/).
The ELISA data analysis was collected SPECTRA Max (450nm) M2e plate reader (Molecular Devices Corp) and processed by SoftMax Pro 5.4. The average (presented asg per ml) and SEM were calculated based on the 4-HNE-BSA standard curves.

For manuscripts utilizing custom algorithms or software that are central to the research but not yet described in published literature, software must be made available to editors and reviewers. We strongly encourage code deposition in a community repository (e.g. GitHub). See the Nature Portfolio guidelines for submitting code & software for further information.

Data

Policy information about availability of data

All manuscripts must include a data availability statement. This statement should provide the following information, where applicable:

- Accession codes, unique identifiers, or web links for publicly available datasets
- A description of any restrictions on data availability
- For clinical datasets or third party data, please ensure that the statement adheres to our policy

The authors declare that data supporting the findings of this study are available within the main text and supplementary materials. The source data underlying Figs. 1a-f, 3k, 4a, 4b, and 5i and Supplementary Figs 2, 3b, 3d, 3e, 5c, 6a, 6b, 6c, 6f, 7g, 8c, and 9c are provided as a Source Data File. All data are available from the corresponding author (chsung@med.cornell.edu) upon reasonable request.

Field-specific reporting

Please select the one below that is the best fit for your research. If you are not sure, read the appropriate sections before making your selection.

☒ Life sciences ☐ Behavioural & social sciences ☐ Ecological, evolutionary & environmental sciences

For a reference copy of the document with all sections, see nature.com/documents/nr-reporting-summary-flat.pdf

Life sciences study design

All studies must disclose on these points even when the disclosure is negative.

Sample size	Preliminary experiments were performed when possible to determine sample size, taking into account resources available and ethical, reductionist animal use.
Data exclusions	No data was excluded from analyses.
Replication	Numbers of the experimental replication or the experiments that were performed independently for each specific result were indicated in the Figure Legends. The number of mice used are described in figure legends and Supplementary Table 1. All attempts of replication were successful.
Randomization	All animals were assigned to groups based on their genotype.
Blinding	The data collection and analyses of mouse experiments and other experiments were blinded. For data collected by objective instruments, such as plate readers and qPCR cyclers and the investigators were not blinded to group allocation during data collection. However, investigator bias is not considered to contribute to the data because the investigator was blinded at the time of data analysis. RNAseq and pathway analyses were performed by a biostatistician who was blinded to experimental groups.

Reporting for specific materials, systems and methods

We require information from authors about some types of materials, experimental systems and methods used in many studies. Here, indicate whether each material, system or method listed is relevant to your study. If you are not sure if a list item applies to your research, read the appropriate section before selecting a response.

Materials & experimental systems

n/a	Involved in the study	
☐	☒	Antibodies
☒	☐	Eukaryotic cell lines
☒	☐	Palaeontology and archaeology
☐	☒	Animals and other organisms
☒	☐	Human research participants
☒	☐	Clinical data
☒	☐	Dual use research of concern

Methods

n/a	Involved in the study	
☒	☐	ChIP-seq
☒	☐	Flow cytometry
☒	☐	MRI-based neuroimaging

Antibodies

Antibodies used

ApoE goat antibody (Millipore #AB947, lot #3385779,).
ApoE goat antibody (Santa Cruz Biotechnique Catalog #sc-6384).
Cre mouse antibody (Millipore #MAB3120, cone 2D8, lot #lv1532663).
Ezrin mouse antibody (Abcam #Ab4069, clone 3C12).
Complement 3 goat antibody (MP Biomedicals #0855444).
Activated C3 rabbit antibody (Hycult Biotech #HM1065, clone 2/11, lot #31650M0621).
CLIC4 rabbit antibody (Homemade affinity-purified CUMC28).
CD45 rat antibody (BD Biosciences #550539, clone 20-F11).
Iba1 rabbit antibody (Wako #019-19741, lot #WEM2922).
Lamp2 rat antibody (DSHB #GL2A7, clone P3U1).
MCT3 rabbit antibody (gift from Dr. Nancy J Philp).
PLVAP rat antibody (DSHB #MECA-32).
PLIN2 rabbit antibody (Proteintech #15294-1-AP).
Vitronectin mouse antibody (Santa Cruz, #sc-74484, clone D-8, lot #C0918).
Vimentin mouse antibody (Dako #M0725, clone V9, lot #M072501049201).
Vimentin mouse antibody (Amersham #RPN1102, batch 21).
Alexa568-conjugated anti-mouse IgG (Thermo Fisher #A10037, lot #1917938).
Alexa 488-conjugated anti-rabbit IgG (Thermo Fisher #A21206).
Alexa 594-conjugated anti-rat IgG (Jackson Immuno Research Labs #712585153).
Alexa 568-conjugated donkey anti-rabbit IgG (Thermo Fisher #A10042).
Alexa 488-conjugated anti-goat IgG (Thermo Fisher #A11055, lot #84B1-1).
Alexa 568-conjugated anti-goat IgG (Thermo Fisher #A11057, lot #1640316).

Validation

Validation of each antibody are listed in Table S2 in Methods.

Animals and other organisms

Policy information about studies involving animals; ARRIVE guidelines recommended for reporting animal research

Laboratory animals

The sex, age, and genotype of the mice used in all experiments are summarized in Table S1. Age of the experimental mice is also listed in Figure Legends. These mice were on a C57BL6/J (Jackson) background as described in the manuscript. Mice were housed in individually ventilated cages (Thoren Caging Systems, Hazelton, PA) on autoclaved aspen-chip bedding (PWI Industries Canada, Quebec, Canada); Cages are changed weekly in either a HEPA-filtered vertical flow change station. γ-irradiated feed (LabDiet 5053, PMI, St Louis, MO, containing24.495% protein,13.122% fat,62.382% Carbohydrates) and acidified reverse osmosis water (pH, 2.5 to 2.8) provided ad libitum. The animal holding room is maintained at a relative humidity of 30 to 70%, the temperature of 21.5 ± 1 ℃, well ventilated, and a 12:12 hour light: dark photoperiod.

Wild animals

The study did not involve wild animals.

Field-collected samples

No field-collected samples were used in this study

Ethics oversight

All procedures using mice were approved by the Weill Medical College of Cornell University Institutional Animal Care and Use Committee.

Note that full information on the approval of the study protocol must also be provided in the manuscript.
